# Cu─O─Al Interfacial Engineering on Cu Nanowires for Durable CO_2_ Electroreduction Into Multi‐Carbon Products

**DOI:** 10.1002/advs.202515557

**Published:** 2025-11-21

**Authors:** Xiaodong Liu, Gang Zhao, Xiaodong Wen, Junyao Wang, Chenchen Hang, Lei Wang, Minliang Lai, Yude Su

**Affiliations:** ^1^ School of Nano Science and Technology State Key Laboratory of Bioinspired Interfacial Materials Science Suzhou Institute for Advanced Research University of Science and Technology of China Suzhou Jiangsu 215123 China; ^2^ School of Chemistry and Materials Science State Key Laboratory of Precision and Intelligent Chemistry University of Science and Technology of China Hefei Anhui 230026 China; ^3^ Institute of Functional Nano and Soft Materials (FUNSOM) Jiangsu Key Laboratory for Carbon‐Based Functional Materials and Devices Soochow University Suzhou 215123 China

**Keywords:** AlOx encapsulation, catalysts stabilization, CO_2_ electroreduction, Cu nanowires, Cu─O─Al interface

## Abstract

The balance between high selectivity and long‐term stability for multi‐carbon (C_2+_) production remains a critical challenge in CO_2_ electrocatalysis due to competing reaction pathways and catalyst reconstruction under operating conditions. In this study, a core‐shell heterostructure is synthesized by encapsulating copper nanowires (Cu NWs) with an aluminum oxide (AlOx) shell. Acting as a Lewis acid, the AlOx shell promotes charge redistribution to stabilize Cu^+^ species at the Cu─O─Al interface while creating an alkaline local microenvironment via ^*^OH adsorption. These effects not only stabilize the catalyst structure but also preserve an optimal ^*^CO intermediate coverage for efficient C─C coupling, as evidenced by in situ Raman spectroscopy and density functional theory (DFT) calculations. As a result, the system achieves a remarkable C_2+_ Faradaic efficiency (FE) of 69.6% at 600 mA cm^−2^ in a flow‐cell configuration. The stability tests further reveal a sustained FE_C2+_ above 50% over 64 h of continuous operation at 300 mA cm^−2^. Tuning of the AlOx shell crystallinity alters product distribution owing to different ^*^OH adsorption capacities at the Cu─O─Al interface. These findings highlight the promise of AlOx encapsulation as a versatile strategy to simultaneously enhance selectivity and durability of Cu‐based catalysts in the electrochemical CO_2_ reduction reaction (eCO_2_RR).

## Graphical abstract

A Cu nanowire/AlOx core‐shell catalyst is reported for CO_2_ electroreduction. Acting as a Lewis acid, the AlOx shell not only stabilizes Cu^+^ species at the Cu─O─Al interface but also creates an alkaline local microenvironment via ^*^OH adsorption. This dual functionality stabilizes the catalyst structure while promoting the C─C coupling process, resulting in efficient and durable multicarbon production.

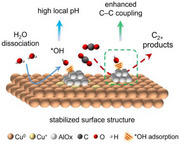

## 1 Introduction

eCO_2_RR offers a promising route for converting CO_2_ into high‐value chemicals under mild conditions.^[^ ^1^, ^2^, ^3^, ^4^ ^]^ However, developing CO_2_‐reducing catalysts that combine high product selectivity and long‐term stability remains a major challenge.^[^ ^5^, ^6^ ^]^ Among the various catalysts explored, Cu‐based materials have shown the highest potential for selectively converting CO_2_ into C_2+_ products.^[^ ^7^, ^8^, ^9^ ^]^ However, their catalytic performance is highly dependent on the local reaction microenvironment, particularly the oxidation state of Cu (Cu^δ+^) and the local pH, which critically influence both product selectivity and catalyst stability.^[^ ^10^, ^11^, ^12^, ^13^ ^]^

Recently, the establishment of a synergistic Cu^0^/Cu^δ+^ couple is considered as an effective strategy to enhance both catalytic selectivity and stability of Cu‐based catalysts in eCO_2_RR.^[^ ^14^, ^15^, ^16^, ^17^, ^18^, ^19^ ^]^ However, the inherent reducibility of Cu^δ+^ species at operational voltages necessitates the development of innovative stabilization strategies to prevent its reduction and subsequent catalyst deactivation. In particular, Cu/oxide interfacial engineering has emerged as a promising approach to stabilize Cu^δ+^ species during eCO_2_RR operation. Oxide materials including SiO_2_, Al_2_O_3_, ZnO, and CeO_2_ have been reported as effective interfacial modulators to anchor the oxidative state of surface Cu atoms for enhanced eCO_2_RR performance.^[^ ^14^, ^20^, ^21^, ^22^, ^23^, ^24^, ^25^, ^26^ ^]^ Among different oxide materials, AlOx has attracted particular attention owing to its simple synthesis, chemical stability, and structural tunability.^[^ ^14^, ^26^, ^27^, ^28^ ^]^ Buonsanti et al. demonstrated that an AlOx shell can lock the Cu surface into an anti‐reduction Cu^2+^ state and thereby suppress the structural reconstruction for stable eCO_2_RR performance.^[^ ^14^ ^]^ Xiong et al. reported a CuAlO_2_ catalyst generated at the Cu/Al_2_O_3_ interface, which can stabilize highly active sites for durable eCO_2_RR operation over 300 h.^[^ ^28^ ^]^ In spite of these research advances, the specific modulation of the interfacial microenvironment by AlOx and the structural optimization of AlOx species in Cu‐based eCO_2_RR remain insufficiently understood and warrant further systematic investigation.^[^ ^12^, ^29^, ^30^, ^31^ ^]^

1D nanowires (NWs) represent a promising class of nanostructures for eCO_2_RR.^[^ ^32^, ^33^, ^34^ ^]^ In this study, Cu NWs were coated with an AlOx shell via a sol–gel method to produce Cu@AlOx_n = 0 or 0.5_ NWs, where n denotes the molar ratio of acetic acid (AA) to aluminum isopropoxide (AIP) during the AlOx synthesis process. The AlOx shell serves as a Lewis acid to facilitate charge redistribution, stabilizing Cu^+^ species at Cu─O─Al interfaces while establishing an alkaline microenvironment through ^*^OH adsorption. This dual functionality improves the catalyst stability and enables optimal ^*^CO intermediate coverage for efficient C─C coupling, as confirmed by in situ Raman characterizations and DFT calculations. As a result, the system achieves a remarkable FE_C2+_ of 69.6% at 600 mA cm^−2^ in a flow‐cell configuration. The stability tests further reveal a sustained FE_C2+_ above 50% over 64 h of continuous operation at 300 mA cm^−2^. Product distribution can also be tuned by adjusting AlOx crystallinity, which modulates the ^*^OH adsorption capacity at Cu─O─Al interfaces. These results identify the mechanistic roles of AlOx encapsulation and highlight its promise as a versatile approach to boost both product selectivity and durability of Cu‐based catalysts in eCO_2_RR.

## 2 Results and Discussion

**Figure 1:**
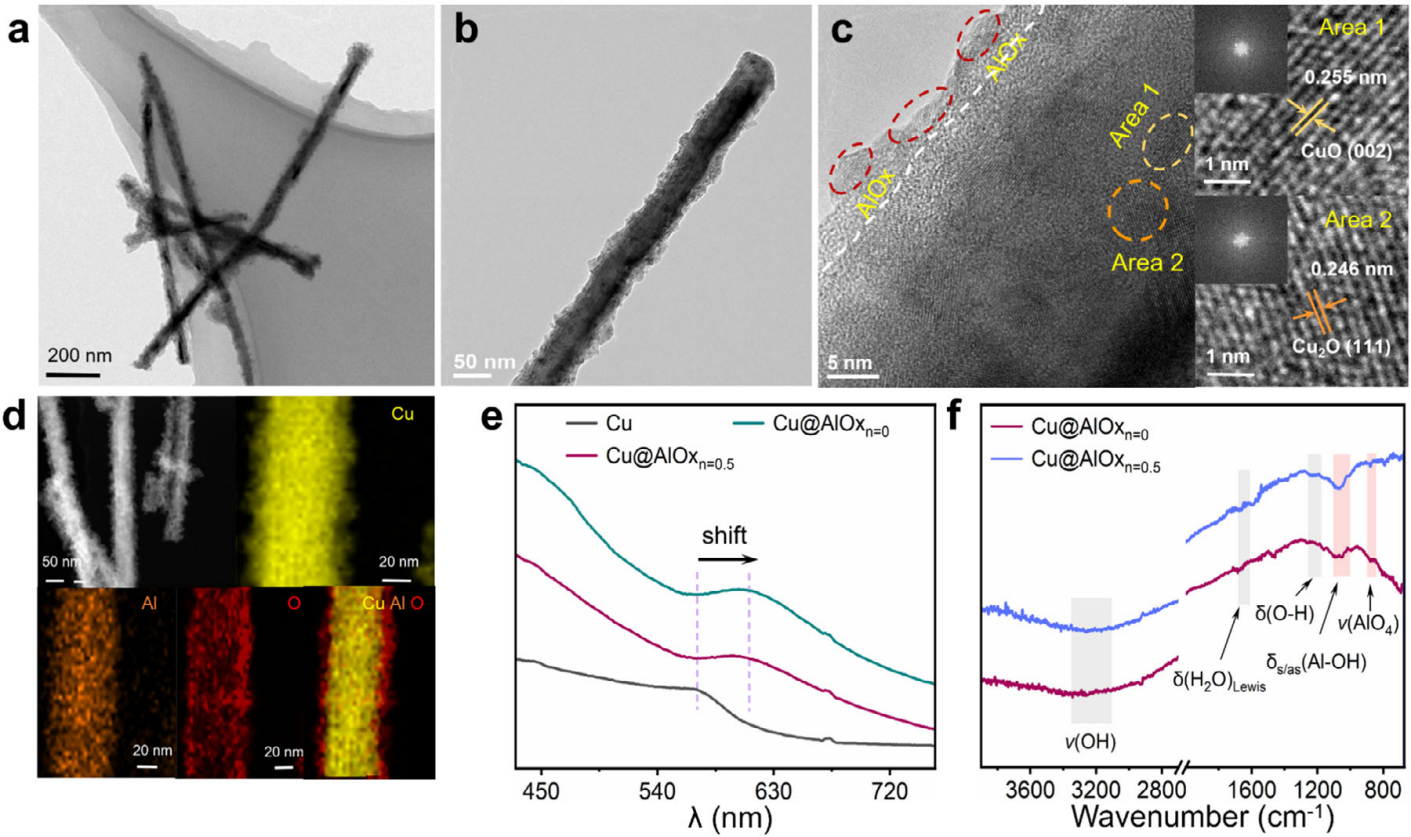
Structural characterizations of pristine Cu and Cu@AlOx NWs before eCO_2_RR measurements. a,b) TEM imaging and c) HRTEM imaging with corresponding fast Fourier transform (FFT) and inverse FFT patterns of Cu@AlOx_n = 0.5_ NWs. d) Representative HAADF‐STEM imaging and corresponding EDS elemental maps of Cu, Al, and their overlap of a representative Cu@AlOx_n = 0.5_ NW. e) UV–vis spectra of pristine Cu and Cu@AlOx NWs. f) FT‐IR spectra of different Cu@AlOx NWs.

Cu NWs were synthesized by adapting a reported protocol with modifications, as detailed in the Supporting Information (SI).^[^ ^32^, ^35^, ^36^ ^]^ Structural characterization by transmission electron microscopy (TEM) and scanning electron microscopy (SEM) confirms the formation of uniform 1D NWs with smooth surfaces (Figures S1 and S2, Supporting Information). The Cu NWs exhibited micron‐scale lengths with an average diameter of 46.7 ± 1.1 nm. Cu@AlOx catalysts with different AlOx shell microstructures (thickness of ≈4.6 nm) were synthesized via controlled hydrolysis of AIP with selective addition of AA while maintaining a fixed H_2_O: AIP molar ratio of 3 (Figures S1 and S3, Supporting Information).^[^ ^37^ ^]^ TEM analysis suggests a core‐shell structure of Cu NWs after AlOx encapsulation, accompanied by a noticeable increase in average diameter from 46.7 to 56.8 nm, enhanced surface roughness, and a reduction in aspect ratios (**Figure** **1**a,b; Figures S1–S4, Supporting Information). High‐resolution TEM (HRTEM) imaging of Cu@AlOx_n = 0.5_ NWs shows lattice spacings corresponding to the (111) and (002) lattice planes of Cu_2_O and CuO, respectively, indicative of specific surface oxidation states of Cu NWs after AlOx encapsulation (Figure 1c; Figure S3, Supporting Information). Elemental mapping performed via energy‐dispersive X‐ray spectroscopy (EDS) in conjunction with high‐angle annular dark‐field scanning transmission electron microscopy (HAADF‐STEM) confirms the relative uniform distribution of Al and O across the nanowire surface (Figure 1d; Figure S4, Supporting Information), consistent with the core‐shell structure observed via TEM imaging (Figure 1a,b). Additionally, UV–vis spectroscopy reveals a characteristic redshift in the surface plasmon resonance upon AlOx coating, attributed to changes in the refractive index surrounding the Cu NWs (Figure 1e).^[^ ^14^ ^]^ The Al content on the Cu NWs was quantified by inductively coupled plasma mass spectrometry (ICP‐MS), yielding an Al: Cu molar ratio of ≈1:34 (Table S1, Supporting Information).

The crystal structures of pristine Cu and Cu@AlOx NWs were characterized by X‐ray diffraction (XRD) analysis (Figure S5, Supporting Information). The XRD patterns confirm that the Cu NWs retain their crystalline structure after AlOx encapsulation. Due to the low AlOx content on Cu NWs, the XRD characteristics of the AlOx shell were examined separately using separate AlOx synthesized without Cu NWs, which exhibited amorphous or boehmite‐like AlO(OH) features (Figure S5b, Supporting Information). The crystallinity of the AlOx shell tends to increase with a reduced n value (AA/AIP molar ratios during the hydrolysis process). Additionally, the Fourier‐transform infrared (FT‐IR) spectra of Cu@AlOx NWs were further analyzed to probe surface functional groups (Figure 1f). The absorption bands at 1635 and 3250 cm^−1^ are attributed to hydroxyl characteristic of boehmite.^[^ ^14^, ^37^ ^]^ Additional bands at 1260, 1050, and 880 cm^−1^ correspond to O─H bending vibrations (δ(O─H)), symmetric and asymmetric bending of Al─OH groups (δ_s/_δ_as_(Al─OH)), and AlO_4_ stretching modes (ν(AlO_4_)), respectively. The AlO_4_ is identified as a Lewis acidic site.^[^ ^14^, ^38^ ^]^ Consequently, FT‐IR spectra exhibit characteristic bands associated with an amorphous/boehmite‐like alumina structure.^[^ ^14^, ^39^ ^]^ Brunauer–Emmett–Teller (BET) analysis reveals microporous features with an average pore size of 2.6 nm (Table S2, Supporting Information), which allows efficient mass transport and product evolution at the solid/liquid/gas interface during eCO_2_RR.

**Figure 2:**
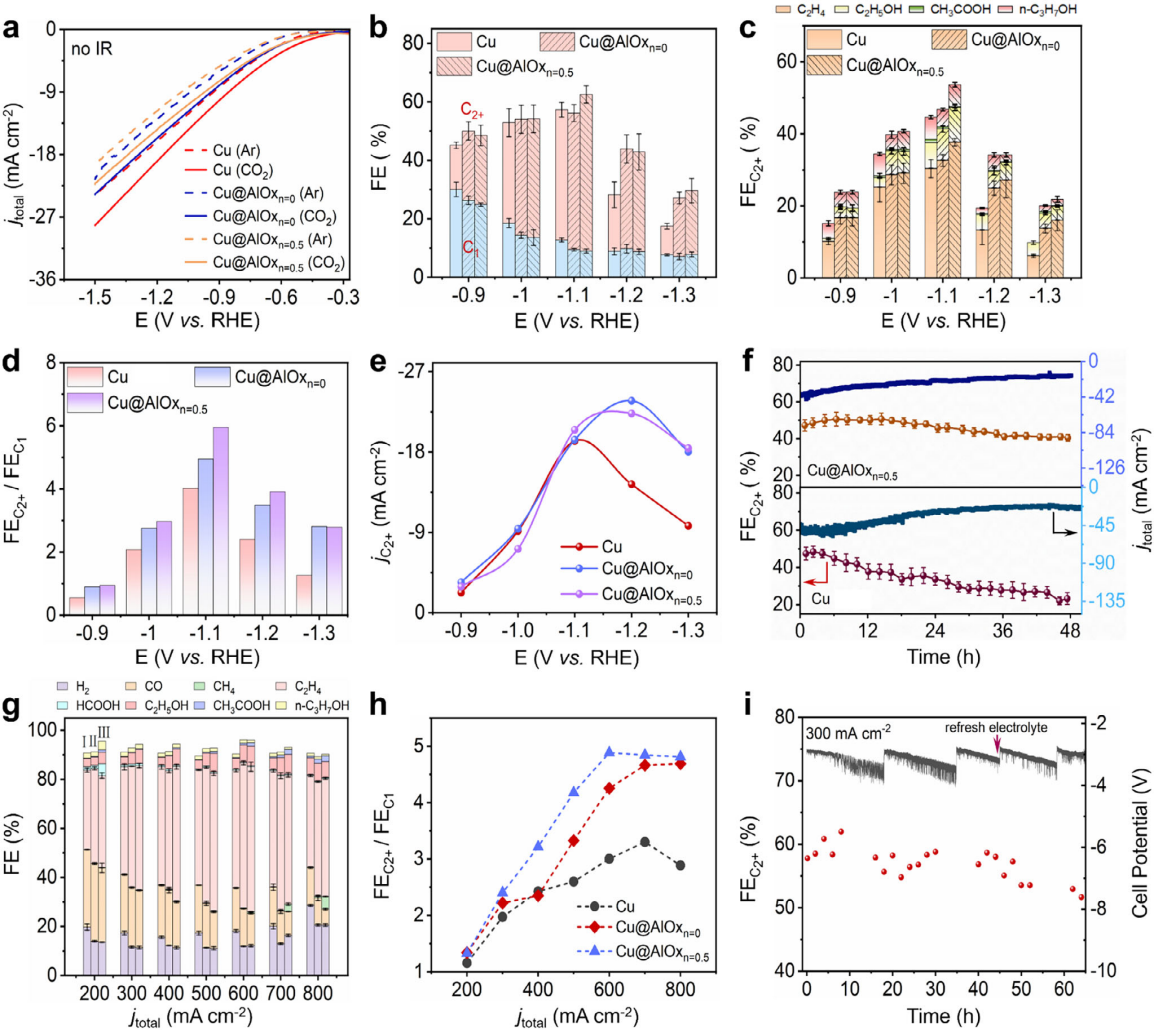
Evaluation of eCO_2_RR performance of pristine Cu and Cu@AlOx NWs. a) LSV curves of pristine Cu, Cu@AlOx_n = 0,_ and Cu@AlOx_n = 0.5_ NWs under Ar‐ and CO_2_‐saturated conditions. b,c) FE of C_1_ and C_2+_ products for pristine Cu, Cu@AlOx_n = 0,_ and Cu@AlOx_n = 0.5_ NWs operating at various applied potentials. d) The ratios of C_2+_/C_1_ products as a function of the applied potential, illustrating the enhanced C_2+_ selectivity of Cu@AlOx NWs. e) Comparison of partial current densities of C_2+_ products at various applied potentials. f) C_2+_ production stability tests of pristine Cu and Cu@AlOx_n = 0.5_ NWs at −1.1 V vs. RHE. The eCO_2_RR measurements in (a‐f) were performed via an H‐cell electrochemical reactor. g) FE of C_1_ and C_2+_ products at various applied current densities in a flow cell reactor with 1.0 m KOH. The numerals I‐III in the figure represent pristine Cu, Cu@AlOx_n = 0,_ and Cu@AlOx_n = 0.5_ NWs, respectively. h) FE_C2+_/FE_C1_ ratios of different catalysts at various applied current densities in a flow cell reactor. i) C_2+_ production stability tests of Cu@AlOx_n = 0.5_ NWs at 300 mA cm^−2^ for 64 h in a flow cell reactor.

The electrocatalytic performance of Cu@AlOx NWs toward eCO_2_RR was first investigated using a standard H‐cell electrochemical reactor with CO_2_‐saturated 0.1 m KHCO_3_ electrolyte. Pristine Cu NWs served as the reference catalyst for comparative analysis. Linear sweep voltammetry (LSV) measurements conducted in both Ar‐saturated (dotted line) and CO_2_‐saturated (solid line) environments (**Figure** **2**a) reveal slightly reduced current densities of Cu@AlOx NWs relative to pristine Cu NWs, which may result from an increased internal resistance of Cu NWs after AlOx encapsulation. Product quantification was performed using online gas chromatography and nuclear magnetic resonance (NMR) spectroscopy (Figures S6 and S7, Supporting Information). As illustrated in Figure 2b, Cu@AlOx NWs exhibit significantly enhanced selectivity toward C_2+_ products compared to pristine Cu NWs within the potential window of −0.9 to −1.3 V vs RHE. At −1.1 V vs RHE, the Cu@AlOx_n = 0.5_ NWs achieve an impressive FE_C2+_ of 54% (Figures 2c,d; S8, Supporting Information). Notably, ethylene (C_2_H_4_) consistently dominated the C_2+_ product distribution, accounting for over 80% of the total C_2+_ selectivity for both pristine Cu and Cu@AlOx NWs (Figures 2c; S8 and S9, and Table S3, Supporting Information). Cu@AlOx NWs synthesized with varying AA/AIP ratios exhibited different product distributions (Figures 2c,d; S8, Supporting Information). Compared to Cu@AlOx_n = 0.5_ NWs, Cu@AlOx_n = 0_ NWs show an overall reduced FE_C2+_. This phenomenon can be explained by the different AlOx crystallinities that alter ^*^OH adsorption capacities, which will be elaborated in the following discussions. Characterizations of the electrochemically active surface area (ECSA) via electrochemical double‐layer capacitance (C_dl_) measurements reveal similar initial ECSA values between Cu@AlOx and pristine Cu NWs (Figures S10–S12 and Table S4, Supporting Information). This observation suggests that the enhanced C_2+_ selectivity of Cu@AlOx NWs may arise from the tailored microenvironment and modified electronic structure at the Cu─O─Al interface, rather than from differences in the available surface area.

The elevated FE_C2+_ of Cu@AlOx_n = 0.5_ NWs directly contributes to enhanced partial current densities for C_2+_ products (*j*_C2+_) (Figure 2e). Chronoamperometric stability tests at −1.1 V vs RHE further demonstrated the remarkable durability of the Cu@AlOx_n = 0.5_ NWs, which sustained an average FE_C2+_ of ≈46% during 48 h of eCO_2_RR operation in an H‐cell reactor (Figure 2f). A similar retention trend in FE_C2+_ and FE_C2H4_ was also observed for Cu@AlOx_n = 0_ NWs (Figures S13,Supporting Information). In contrast to the sustained FE_C2+_ of Cu@AlOx NWs, pristine Cu NWs exhibit significantly declined eCO_2_RR performance over time, with FE_C2+_ dropping from 50% to 18% within 48 h, presumably due to uncontrolled surface reconstruction that gives rise to catalyst degradation. To investigate the impact of AlOx thickness on the FE_C2+_ stability of Cu@AlOx NWs, we further reduced the thickness of the AlOx coating on Cu@AlOx_n = 0.5_ NWs from ≈4.6 to ≈1.6 nm (thin‐Cu@AlOx_n = 0.5_ NWs, Figure S14, Supporting Information). Stability test of thin‐Cu@AlOx_n = 0.5_ NWs shows that the FE_C2+_ tends to decrease from 45.4% to 24.3% within 48 h (Figure S15, Supporting Information). This declining trend is similar to that of pristine Cu NWs, in contrast to the stable FE_C2+_ observed for the Cu@AlOx_n = 0.5_ NWs. These results demonstrate that an AlOx encapsulation layer with a thickness of ≈5 nm is essential to stabilize the Cu NWs against performance degradation over time.

In order to evaluate the eCO_2_RR performance of both pristine Cu and Cu@AlOx NWs at industrial‐relevant current densities, we further performed eCO_2_RR measurements using a flow cell reactor at various current densities ranging from 200 to 800 mA cm^−2^. As demonstrated in Figure 2g, Cu@AlOx_n = 0_ and Cu@AlOx_n = 0.5_ NWs show consistently higher FE_C2+_ than the pristine Cu NWs across the full current density range. In particular, at 600 mA cm^−2^, Cu@AlOx_n = 0.5_ NWs show a remarkable FE_C2+_ of 69.6%, significantly outperforming the pristine Cu NWs (FE_C2+_ of 54.2%). The ratios of C_2+_/C_1_ products for Cu@AlOx_n = 0.5_ and Cu@AlOx_n = 0_ NWs reach 4.9 and 4.3 at 600 mA cm^−2^ (Figure 2h), which are both significantly superior to that of pristine Cu NWs (C_2+_/C_1_ ratio of 3.0). It should be noted that the improvement in FE_C2+_ after AlOx encapsulation is more significantly demonstrated in a flow cell reactor (15.4% improvement) in comparison with a H‐cell reactor (less than 10% improvement). This is attributed to the sufficient CO_2_ supply in a flow cell setup, which unlocks the full benefits of the AlOx coating for promoted C─C coupling. We further evaluated the stability of FE_C2+_ during long‐term eCO_2_RR operation (at 300 mA cm^−2^) in a flow cell configuration. As illustrated in Figure 2i, Cu@AlOx_n = 0.5_ NWs maintain a stable FE_C2+_ above 50% over 64 h of flow cell measurements. In contrast, FE_C2+_ of the pristine Cu NWs dramatically declines from 58.2% to 18.9% over 10 h of flow cell measurements (Figure S16, Supporting Information). The trend in catalyst stability observed via a flow cell is consistent with that observed in an H‐cell reactor, demonstrating the significantly enhanced durability of Cu NWs after AlOx encapsulation. In comparison with the state‐of‐the‐art Cu NW catalysts reported in previous literature, the Cu@AlOx_n = 0.5_ NWs demonstrate comparable selectivity and stability toward C_2+_ production in both H‐cell and flow cell configurations (Table S5, Supporting Information).

**Figure 3:**
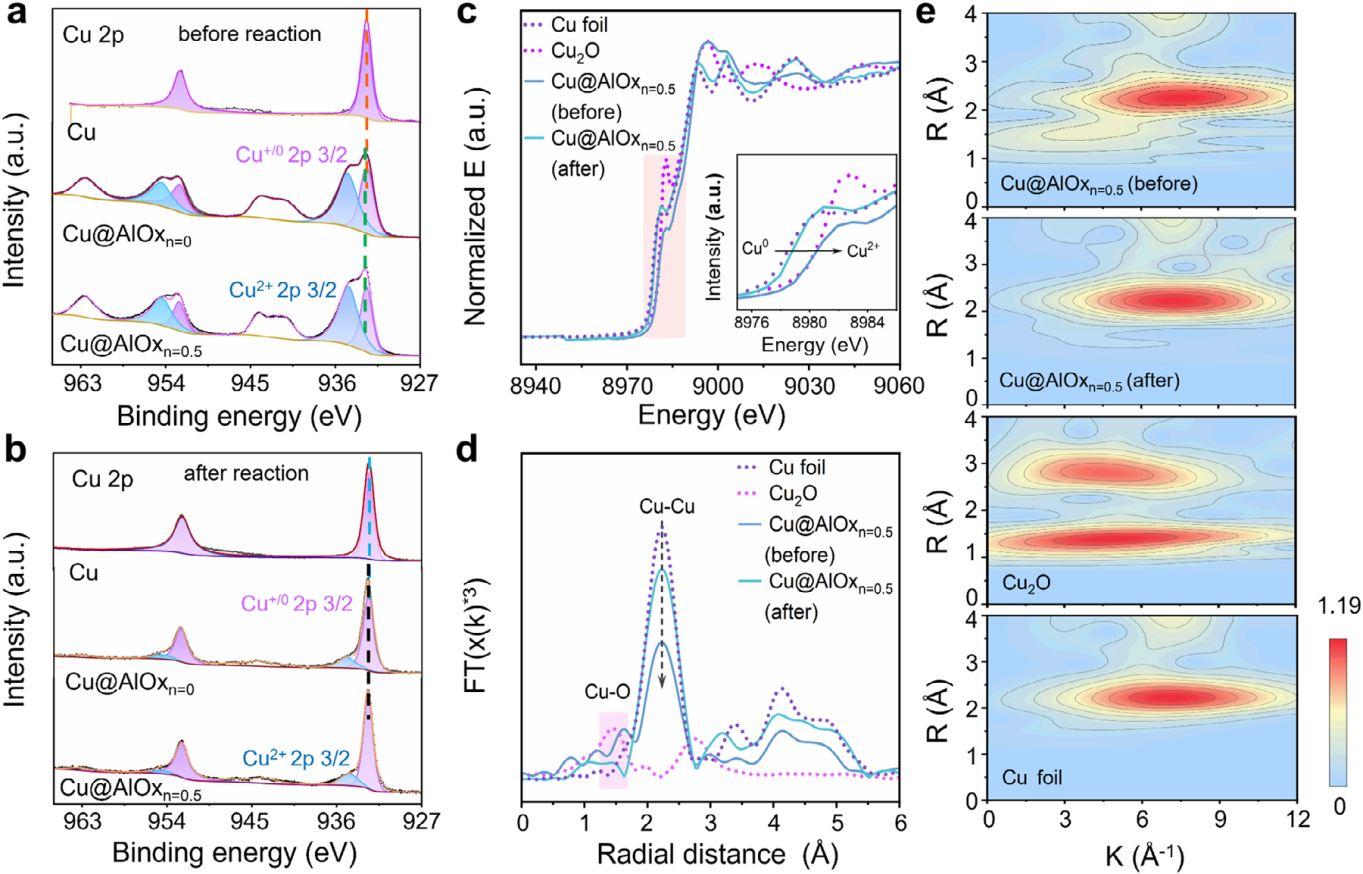
Chemical structural characterizations of pristine Cu and Cu@AlOx NWs before and after eCO_2_RR measurements at −1.1V vs. RHE for 24 h in an H‐cell reactor. Cu 2p XPS spectra a) before and b) after eCO_2_RR measurements. c) Cu K‐edge XANES spectra, with an inset showing an enlarged region (8975–8986 eV). d) Cu K‐edge FT‐EXAFS spectra. e) Wavelet transform of the k^3^‐weighted EXAFS data, highlighting Cu─O and Cu─Cu bond coexistence in Cu@AlOx_n = 0.5_ NWs during eCO_2_RR measurements.

Understanding the catalytic structure‐activity relationship is critical for elucidating the intrinsic mechanisms governing eCO_2_RR. To elucidate the evolution of surface valence states and chemical compositions in both pristine Cu and Cu@AlOx NWs, a comprehensive X‐ray photoelectron spectroscopy (XPS) analysis was conducted. **Figure** **3**a presents the Cu 2p XPS spectra of pristine Cu, Cu@AlOx_n = 0_, and Cu@AlOx_n = 0.5_ NWs prior to eCO_2_RR measurements, while supplementary Figures S17 and S18 (Supporting Information) provide additional Al 2p and O 1s spectra information. Notably, Cu^0/+^ features are clearly observed in pristine Cu NWs before eCO_2_RR operation (Figure 2a). The presence of Cu^+^ species is attributed to the spontaneous oxidation of surface Cu atoms during the surfactant removal step in the purification process.^[^ ^32^, ^40^, ^41^ ^]^ Due to the AlOx encapsulation process being carried out under non‐inert conditions, distinct Cu^2+^ signals were detected following AlOx deposition on Cu NWs (Figure 2a). It should also be noted that the Cu 2p peak shifts from 932.66 eV for pristine Cu NWs to 932.78 eV for Cu@AlOx_n = 0.5_ NWs. This peak shift may result from electron redistribution from Cu to AlOx owing to the Lewis acid nature of the AlOx coating (Table S6, Supporting Information).

In order to understand the active oxidation state of Cu during eCO_2_RR, the post‐reaction Cu NWs were further analyzed by XPS. In order to minimize the impact of post‐reaction reoxidation of surface Cu species, all catalysts were promptly transferred into a vacuum bag following the eCO_2_RR operation for consistency. The Cu 2p XPS spectra reveal a dominant coexistence of Cu^+^/Cu^0^ species in the Cu@AlOx NWs, while Cu^2+^ is significantly reduced (Figure 2b). The residual Cu^2^⁺ signals are likely attributed to surface oxidative states induced by electronic redistribution on Cu─O─Al interface.^[^ ^32^ ^]^ Cu LMM Auger electron spectroscopy further corroborated these findings (Figure S19 and Table S7, Supporting Information). In contrast, pristine Cu NWs exhibit dominating Cu^0^ species following eCO_2_RR, indicating the reduction of most Cu^+^ species during the electrochemical reaction. Under electroreduction conditions, the structural integrity of the Cu─O─Al interface in Cu@AlOx NWs was further confirmed by the sustained presence of lattice oxygen (Cu─O_latt_) and Al element (Figures S17 and S18 and Table S8, Supporting Information).^[^ ^14^, ^42^ ^]^ The Al 2p binding energies exhibit slight variations between Cu@AlOx_n = 0_ and Cu@AlOx_n = 0.5_ samples, suggesting different AlOx coordination environments (Table S8, Supporting Information). Such a difference may influence the microenvironment at Cu─O─Al interfaces and thereby alter the product selectivity in eCO_2_RR.

Complementary X‐ray absorption spectroscopy (XAS) analyses provide further insights into the electronic structure evolution of the Cu@AlOx NWs. Here, Cu@AlOx_n = 0.5_ NWs were selected as representative samples for XAS analyses owing to their superior FE_C2+_ during eCO_2_RR measurements. X‐ray absorption near‐edge structure (XANES) spectra revealed the presence of Cu^2+^/Cu^+^ species prior to reaction,^[^ ^43^ ^]^ with significant reduction of Cu^2+^ observed after eCO_2_RR measurements, while Cu^+^ species remained preserved (Figure 3c). Extended X‐ray absorption fine structure (EXAFS) fitting reveals characteristic Cu─O and Cu─Cu coordination distances of ≈1.5 and 2.1 Å, respectively, prior to eCO_2_RR measurements (Figure 3d). After eCO_2_RR measurements, a slight contraction in the Cu─O bond length was observed (ΔR ≈ 0.1 Å), indicating small changes in the local coordination environment during eCO_2_RR operation. EXAFS fitting confirmed the coexistence of Cu─O and Cu─Cu coordination environments in the post‐reaction Cu@AlOx_n = 0.5_ NWs (Figure S20, Supporting Information). The simultaneous presence of Cu^0^ and Cu^+^ species was further corroborated by wavelet transform analysis (Figure 3e). The persisted Cu─O bond features in the EXAFS spectrum further support the preservation of Cu^+^ species during eCO_2_RR operation, while the reduced intensities are attributed to the reduction of Cu^2+^ species. Overall, XPS and XAS analyses demonstrate the presence of a Cu─O─Al interface that effectively stabilizes the Cu⁺ species during eCO_2_RR operation. This stabilization of Cu⁺ species is further supported by cyclic voltammetry (CV) measurements, which reveal characteristic redox features corresponding to the reduction of Cu^δ+^ species (Figure S21, Supporting Information). The reduction peak corresponding to the oxidized Cu species in the Cu@AlOx NWs was shifted to a more negative potential compared to that of pristine Cu NWs, indicating a hindered Cu^+^‐to‐Cu^0^ reduction process in the presence of AlOx shell.

The sustained presence of Cu⁺ species plays a key role in stabilizing the structure of Cu NWs and thereby the eCO_2_RR performance over extended electrocatalytic operation. Previous studies suggest that Cu‐based catalysts typically undergo dynamic reconstruction during eCO_2_RR, which is driven by atomic migration and the redox‐mediated dissolution/reprecipitation of surface Cu atoms under electroreduction conditions.^[^ ^14^, ^24^, ^44^ ^]^ The preservation of Cu^+^ species and thereby the formation of Cu^+^─O─Al linkages on Cu@AlOx contribute to anchoring the valence state of surface Cu, significantly suppressing the redox‐mediated dissolution/reprecipitation processes.^[^ ^14^ ^]^ The Cu─O─Al interface can also enhance the surface adhesion energy of Cu atoms, thereby reinforcing structural integrity.^[^ ^14^, ^43^ ^]^ An insufficient Cu─O─Al interfacial density resulting from a reduced initial AlOx thickness (≈1.6 nm) may compromise the stability of the Cu@AlOx NWs, as evidenced by the eCO_2_RR stability test of thin‐Cu@AlOx_n = 0.5_ NWs shown in Figure S15 (Supporting Information). The structural integrity Cu@AlOx NWs during eCO_2_RR measurements is confirmed by post‐reaction TEM and XRD analyses, which show both preserved morphological and crystallographic structure after prolonged continuous operation (Figures S22 and S23,Supporting Information). In contrast, we observed pronounced structural degradation of pristine Cu NWs after 24 h of eCO_2_RR operation, as evidenced from post‐reaction TEM analyses and the change in their ECSA (Figures S10–S11, S22 and Table S4, Supporting Information). The structural preservation of Cu NWs is closely correlated with the retention of FE_C2+_ during eCO_2_RR stability tests (Figures 2f,i; S13 and S16, Supporting Information). Previous literature suggests that reconstructed rough facets and fragmented small particles of Cu electrocatalysts generate isolated low‐coordinated Cu sites, which can favor HER instead of eCO_2_RR.^[^ ^22^, ^33^, ^45^ ^]^ As a result, the structural integrity of Cu@AlOx NWs, which stems from the preservation of Cu^+^ at the Cu─O─Al interface, determines their superior retention of FE_C2+_ over prolonged operation. Post‐reaction Al 2p XPS and ICP analysis (Tables S8 and S9, Supporting Information) both reveal partial degradation of the AlOx layer in Cu@AlOx NWs over extended eCO_2_RR measurements, which correlates with the slight decline in FE_C2+_ after prolonged operation (Figures 2f,i; S13, Supporting Information). The partial degradation of the AlOx layer is attributed to the enhanced local alkaline environment for Cu@AlOx NWs, which will be elaborated in following discussions.

**Figure 4:**
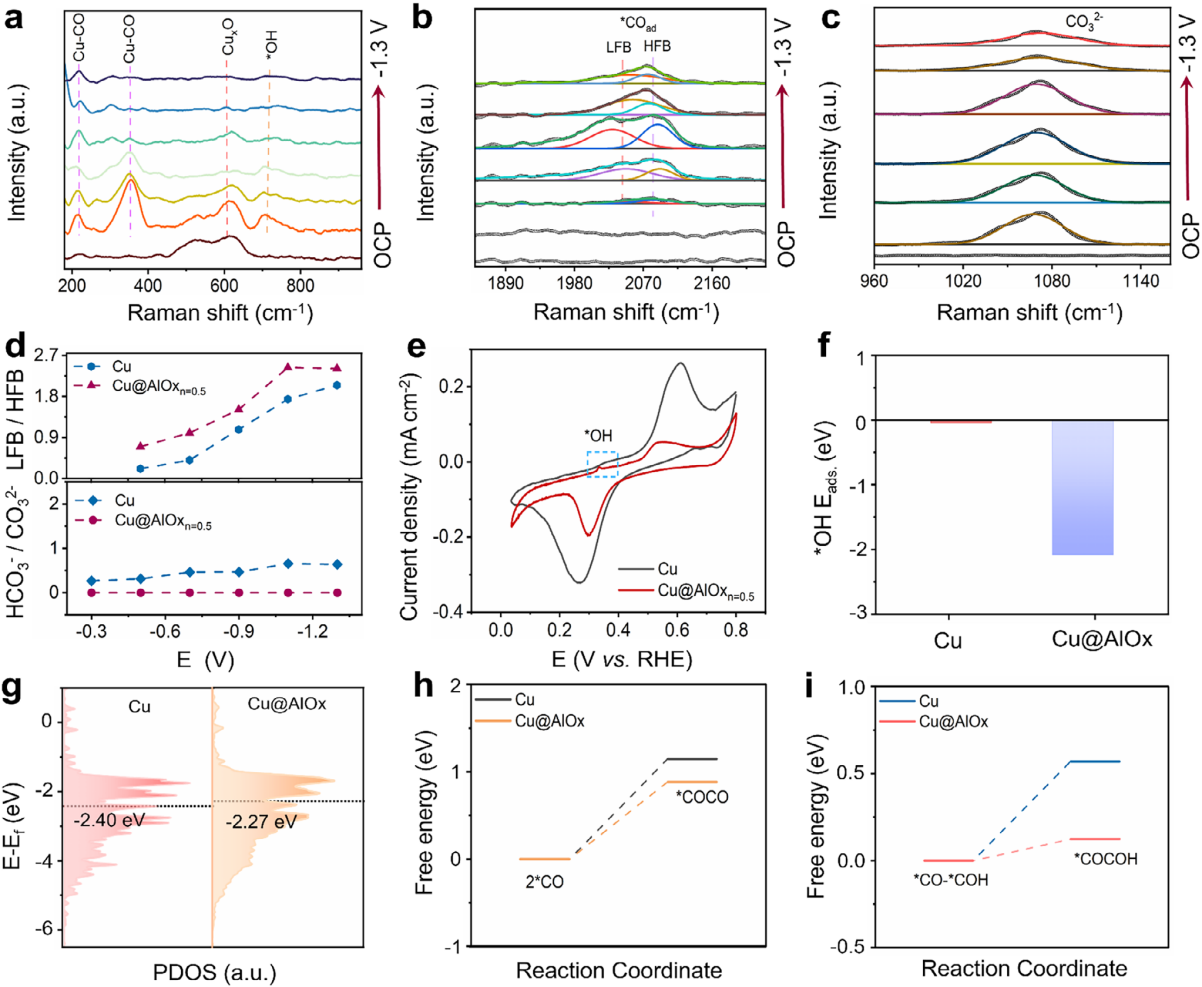
a,b,c) In situ Raman spectra and corresponding fittings of Cu@AlOx_n = 0.5_ NWs at a series of applied potentials (from OCP to −1.3 V vs. RHE) in CO_2_‐saturated 0.1m KHCO_3_. d) The LFB/HFB ratio (upper panel) and HCO_3_^−^/CO_3_^2−^ ratio (lower panel) as a function of the applied potential. e) CV curves of ^*^OH for pristine Cu and Cu@AlOx_n = 0.5_ NWs in 0.1m KOH. f) The calculated adsorption energy of ^*^OH on pristine Cu and Cu@AlOx. g) PDOS diagrams of pristine Cu and Cu@AlOx. h) The calculated free energy barrier of 2^*^CO to ^*^OCCO on pristine Cu and Cu@AlOx. i) The calculated free energy barrier of ^*^CO─^*^COH to ^*^COCOH on pristine Cu and Cu@AlOx.

To further elucidate the superior eCO_2_RR performance of Cu@AlOx NWs, in situ Raman spectroscopy was performed to monitor the real‐time evolution of surface Cu species and key surface‐bound intermediates during eCO_2_RR operation. Again, Cu@AlOx_n = 0.5_ NWs were selected as the representative samples for in situ Raman analyses, with pristine Cu NWs as the reference. Interestingly, Cu@AlOx_n = 0.5_ NWs exhibit persistent Cu─O Raman signals at 521 and 620 cm^−1^ across a broad potential range from open‐circuit potential (OCP) to −1.3 V vs RHE (**Figures** **4**a; S24, Supporting Information). These sustained Raman characteristics stand in sharp contrast to the rapid Cu^+^‐to‐Cu^0^ reduction observed in pristine Cu NWs (Figure S25, Supporting Information), highlighting the enhanced retention of oxidized Cu species in the presence of AlOx coating during eCO_2_RR operation. These findings are consistent with the XPS and XAS results, further indicating the key roles of Cu─O─Al interfacial structure in stabilizing Cu⁺ species.^[^ ^29^, ^30^, ^41^ ^]^

Next, we move forward to analyze the ^*^CO signals from the in situ Raman spectra. As shown in Figures 4a,b and S24 and S25 (Supporting Information), the peaks at 220 and 361 cm^−1^ wavenumber regions are attributed to the stretching and rotational vibrational modes of the Cu─CO bond, respectively.^[^ ^46^ ^]^ The more intense ^*^CO signals observed for the Cu@AlOx_n = 0.5_ NWs indicate a higher ^*^CO surface coverage, which facilitates subsequent C─C coupling processes.^[^ ^47^ ^]^ Additionally, the C≡O stretching vibration of adsorbed ^*^CO manifests as a peak at 2064 cm^−1^ in particular under large overpotentials (−0.7 to −1.3 V vs RHE), which can be deconvoluted into two distinct components: a low‐frequency C≡O stretching band (LFB) and a high‐frequency C≡O stretching band (HFB). In particular, the LFB is attributed to dynamically absorbed ^*^CO intermediates that actively participate in the subsequent C─C coupling steps.^[^ ^15^, ^47^, ^48^, ^49^, ^50^ ^]^ The increased intensities of the LFB signal for Cu@AlOx_n = 0.5_ NWs, compared to pristine Cu NWs (Figures 4b,d; S25b, Supporting Information), indicate enhanced stabilization of dynamic ^*^CO intermediates, which promote the formation of C_2+_ product.^[^ ^30^, ^41^ ^]^ These findings emphasize the critical role of the Cu─O─Al interfacial structure in enhancing the surface ^*^CO coverage and facilitating multi‐carbon production during eCO_2_RR operation.

Additionally, a distinct Raman peak ≈710 cm^−1^, attributed to adsorbed ^*^OH, was observed in both pristine Cu and Cu@AlOx_n = 0.5_ NWs.^[^ ^29^ ^]^ However, the ^*^OH peak intensity on Cu@AlOx_n = 0.5_ NWs was much higher than that on pristine Cu NWs (Figures 4a; S24 and S25, Supporting Information). Additionally, Cu@AlOx_n = 0.5_ NWs exhibit a negligible HCO_3_^−^ peak but a notable CO_3_^2−^ peak at 1030–1070 cm^−1^, in contrast to the significant HCO_3_^−^ peak with a considerable HCO_3_^−^/CO_3_^2^^−^ peak area ratio (0.27–0.64) on pristine Cu NWs (Figures 4c,d; S25c, Supporting Information). Since the HCO_3_^−^/CO_3_^2^^−^ peak area ratio is directly correlated with the local OH^−^ concentration, the dominance of CO_3_^2−^ on Cu@AlOx_n = 0.5_ NWs clearly demonstrates an enhanced local alkalinity after AlOx coating. To further clarify the role of AlOx sites in enhancing ^*^OH adsorption, hydroxide (^*^OH) electro‐adsorption analysis was further conducted. As shown in Figures 4e, Cu@AlOx_n = 0.5_ NWs exhibit a much stronger ^*^OH adsorption signal relative to pristine Cu NWs, indicating an enriched ^*^OH species in the presence of AlOx encapsulation. Previous studies suggest that metal oxides can serve as Lewis acid sites to adsorb the ^*^OH species produced from water dissociation, resulting in a higher ^*^OH concentration at the Cu/oxide interface.^[^ ^12^ ^]^ In addition, we further compared the ^*^OH adsorption characteristics between Cu@AlOx_n = 0_ and Cu@AlOx_n = 0.5_ samples. We found that the ^*^OH adsorption peak intensity of Cu@AlOx_n = 0.5_ is stronger than that of the Cu@AlOx_n = 0_ counterpart (Figure S26, Supporting Information). Based on the XRD analysis (Figure S5, Supporting Information), AlOx_n = 0.5_ shows a reduced crystallinity compared to AlOx_n = 0_. A more amorphous AlOx structure generates abundant unsaturated Al coordination sites, which may provide more Lewis acid sites for enhanced ^*^OH adsorption. The enhanced ^*^OH adsorption results in an elevated local pH at the Cu surface, thereby enriching the local CO_2_ concentration and potentially reducing the kinetic barrier for the hydrogenation of key intermediates.^[^ ^12^ ^]^ Altogether, these results support the trend in FE_C2+_ (Cu@AlOx_n = 0.5_ > Cu@AlOx_n = 0_ > pristine Cu NWs) observed in eCO_2_RR measurements (Figures 2; S8, Supporting Information). In addition to the benefits in promoting C─C coupling, the enhanced local alkaline environment may facilitate the degradation of the AlOx coating, as evidenced from the post‐reaction XPS and ICP analyses (Tables S8 and S9, Supporting Information). More advanced AlOx synthetic approaches such as colloidal atomic layer deposition may produce a more robust AlOx shell with higher alkaline tolerance,^[^ ^14^ ^]^ which will be the focus of our future work.

In order to provide a theoretical basis for the enhanced FE_C2+_ of the Cu@AlOx NWs, we performed additional DFT calculations (Figures 4f–i; S27–S29, Supporting Information). First, DFT calculations of the ^*^OH adsorption energy show a more negative value for Cu@AlOx compared to pristine Cu (Figure 4f). This calculation result is consistent with the enhanced ^*^OH adsorption for Cu@AlOx NWs observed via in situ Raman and electro‐adsorption characterizations mentioned above (Figure 4a–e). Projected density of states (PDOS) analysis reveals that the d‐band center of Cu@AlOx (−2.27 eV) shifts toward the Fermi level compared to pristine Cu (−2.40 eV), suggesting reduced energy barrier of CO_2_ reaction and accelerated electron transfer (Figure 4g).^[^ ^12^, ^29^ ^]^ Furthermore, the upshift of the *d*‐band center can enhance the substrate‐adsorbent interaction, especially the adsorption of ^*^CO on Cu@AlOx surface, consistent with the enhanced ^*^CO coverage of Cu@AlOx_n = 0.5_ NWs observed in in situ Raman characterizations. In order to further understand the reaction pathways leading to C_2+_ products, the transition state barriers for two typical C─C coupling mechanisms via ^*^CO and ^*^COH were calculated: ^*^CO‐^*^CO and ^*^CO─^*^COH conversion on Cu@AlOx (with the presence of Cu^+^/Cu° couple) and pristine Cu surfaces (without the presence of Cu^+^), respectively (Figures 4h,i; S27–S29, Supporting Information). While ^*^CO‐^*^CO coupling faces significant energy barriers on both Cu@AlOx (0.88 eV) and pristine Cu surfaces (1.15 eV) (Figure 4h), ^*^CO─^*^COH coupling is energetically more favorable (Figure 4i, 0.12 eV for Cu@AlOx and 0.57 eV for pristine Cu). For both ^*^CO‐^*^CO and ^*^CO─^*^COH pathways, our calculations show that the presence of AlOx encapsulation can reduce the energy barrier for C─C coupling, which results from the presence of Cu^+^ species at the Cu─O─Al interface that account for electron redistribution. These theoretical calculation results are consistent with the in situ Raman results and the superior FE_C2+_ of Cu@AlOx NWs observed in eCO_2_RR measurements. While the enhanced local alkalinity and the stabilized Cu^+^ at the Cu─O─Al interface both contribute to promoting the C─C coupling process, these two factors function in different ways: the local alkaline environment enhances C─C coupling by enriching local CO_2_ concentration and potentially promoting the hydrogenation of key intermediates,^[^ ^12^ ^]^ whereas Cu⁺ stabilization directly governs the coupling efficiency via electronic modulation of the surface Cu sites. The Cu─O─Al interface provides the structural foundation that enables the synergistic interplay between these two aspects, rendering the durable C_2+_ production of the Cu@AlOx NWs.

## 3 Conclusion

In this study, we present an AlOx encapsulation approach to simultaneously enhance the product selectivity and operational stability of Cu NW catalysts in eCO_2_RR. The AlOx encapsulation creates a robust Cu─O─Al interface that not only stabilizes surface Cu^+^ species but also generates an alkaline local microenvironment via ^*^OH adsorption. This dual functionality results in a superior catalyst stability and an enhanced ^*^CO intermediate coverage for efficient C─C coupling. As a consequence, the Cu@AlOx NWs achieve an optimal FE_C2+_ of 69.6% at 600 mA cm^−2^ in a flow‐cell configuration, along with a sustained FE_C2+_ above 50% over 64 h of continuous operation at 300 mA cm^−2^. In contrast, unencapsulated pristine CuNWs show structural deterioration with dramatically declined eCO_2_RR activity under identical stability testing conditions. We also demonstrate that the product distribution can be further tuned by adjusting the AlOx crystallinity to modulate ^*^OH adsorption capacity at Cu─O─Al interfaces. Our results highlight AlOx coating as a versatile approach to concomitantly enhance the product selectivity and durability of Cu‐based catalysts for efficient eCO_2_RR.

## Conflict of Interest

The authors declare no conflict of interest.

## Author Contributions

Y.S. conceived the ideas and designed the experiments. X.L. contributed to material synthesis and characterizations, electrochemical measurements, and data analyses. G.Z. and L.W. contributed to XAS analysis and material characterizations. X.W. assisted in material synthesis. J.W. assisted in material characterizations. C.H. and M.L. assisted in manuscript revision. X.L. and Y.S. wrote the manuscript. All authors contributed to discussions and manuscript review.

## Supporting information



Supporting Information

## Data Availability

The data that support the findings of this study are available from the corresponding author upon reasonable request.
