# Supplementary material for: Cu─O─Al Interfacial Engineering on Cu Nanowires for Durable CO2 Electroreduction Into Multi‐Carbon Products
Source: Adv Sci (Weinh). 2025 Nov 21;13(8):e15557. doi: 10.1002/advs.202515557 (PMC12884756; doi:10.1002/advs.202515557)
Supplement: Supplementary file 1 — Supporting Information [file ADVS-13-e15557-s001.docx]

**Supporting Information**

**Cu–O–Al Interfacial Engineering on Cu Nanowires for Durable CO_2_ Electroreduction into Multi-carbon Products**

Xiaodong Liu ^a^, Gang Zhao ^a,b^, Xiaodong Wen ^a,b^, Junyao Wang ^a,b^, Chenchen Hang ^a^, Lei Wang ^c^, Minliang Lai ^a,b^*, Yude Su ^a,b^*

^a^ School of Nano Science and Technology, State Key Laboratory of Bioinspired Interfacial Materials Science, Suzhou Institute for Advanced Research, University of Science and Technology of China, Suzhou, Jiangsu 215123, China

^b^ School of Chemistry and Materials Science, State Key Laboratory of Precision and Intelligent Chemistry, University of Science and Technology of China, Hefei, Anhui 230026, China

^c^ Institute of Functional Nano and Soft Materials (FUNSOM), Jiangsu Key Laboratory for Carbon-Based Functional Materials and Devices, Soochow University, Suzhou 215123, China

**Experimental Section**

**Chemicals and Materials.** Copper chloride dihydrate (CuCl_2_∙2H_2_O, 99%), D-(+)-glucose (C_6_H_12_O_6_, 99.5%), hexadecylamine (HDA, 98%), potassium hydrogen carbonate (KHCO_3_, 99.7%), aluminum isopropoxide (AIP, 99.8%) and 5% Nafion solution were all purchased from Aladdin. Ethanol (200 proof), isopropanol (IPA), acetic acid (AA, 99%), and n-hexane were obtained from Sinopharm Group. We used deionized (DI) water with a resistivity of 18.2 MΩ·cm at room temperature for all the experiments.

**Synthesis of Cu NWs.** The pristine Cu nanowires (Cu NWs) were prepared by following a previously reported protocol.^[1, 2]^ Briefly, 60 mL of an aqueous solution containing 136 mg CuCl_2_∙2H_2_O, 770 mg HDA, and 173 mg D-(+)-glucose was magnetically stirred 12h to form a light-blue mixture, followed by sonication for 20 min. Then the solution was transferred to a Teflon-lined autoclave and heated to 120 °C for 16 h. The solid product was collected by centrifugation at 3500 rpm, washed three times with a hexane/ethanol (1:1 v/v) mixture and twice with ethanol (5 min per cycle). The Cu NWs were stored in Ar-saturated ethanol solution.

**Synthesis of Cu@AlOx NWs.** Cu@AlOx NWs were synthesized using AIP as the aluminum precursor, AA as the hydrolysis rate controller, and IPA as the solvent. Initially, the Cu NWs were dispersed in IPA under sonication for 10 min. A separate solution of AIP in IPA (25 mM) was prepared under continuous stirring. The mass ratio of Cu NWs to AIP is fixed at 9.4:1. Water was added to the AIP solution at a controlled molar ratio of 3:1, and the AA/AIP molar ratio was varied from 0 to 0.5 (named Cu@AlOx_n=0_, Cu@AlOx_n=0.5_), in which n represents the rate of AA/AIP. Then the gel was further aged at 60°C in an incomplete inert atmosphere for 20 h under reflux and 350 rpm magnetic stirring. The final products were washed with ethanol several times and finally dried in vacuum at 70°C for 12 h.^[3-5]^ Cu@AlOx_n=0.5_ NWs with a thinner AlOx layer (thin-Cu@AlOx_n=0.5_) were prepared following the same procedure as Cu@AlOx_n=0.5_, where the mass ratio of Cu NWs to AIP was maintained at 37.6:1. In the synthesis, the water-to-AIP molar ratio was fixed at 3:1, while the AA/AIP ratio was set to 0.5.

**Synthesis of AlOx.** AlOx was synthesized under the same reaction conditions as the synthesis of Cu@AlOx NW without adding Cu NWs.

**Characterizations**: Transmission electron microscope (TEM) was performed using Tecnai G2 F20 S-TWIN and JEOL JEM-2100F. The crystalline structures were analyzed by X-ray diffraction (XRD) on a Rigaku D/max-2500 X-ray diffractometer with Cu-Kα radiation. Elemental analysis was conducted using X-ray photoelectron spectroscopy (XPS, Thermo Fisher Scientific with Al Kα X-ray resource). Inductively coupled plasma mass spectrometry (ICP-MS) was performed on the Agilent 5110 model to determine the Cu and Al concentration in Cu@AlOx NW. To avoid oxidation before characterization, all the electrocatalysts were washed with ethanol post-electrocatalysis, dried under vacuum, and finally stored in sealed vacuum bags.

**Electrochemical measurements.** Typically, the catalyst ink was prepared by dispersing 5 mg of catalyst and 50 µL of Nafion (5%) in 950 mL of ethanol under sonication for 30 min. Then 60 uL of ink was dropped onto hydrophobic carbon paper (1×1cm^2^) and dried for the subsequent electrochemical tests. Electrochemical CO_2_RR measurements were carried out in an H-type cell with a three-electrode system separated by an anion-exchange membrane (AEM, Fumasep FAA-3-PK-75), and tested by Biologic multichannel electrochemical workstation (VSP s/n:2406, Biologic). Each compartment holds 35 mL of 0.1 M KHCO_3_ aqueous solution saturated with CO_2_. Before the experiments, the electrolyte in the cathodic compartment was saturated with CO_2_ by bubbling CO_2_ gas for at least 30 min. During the reduction experiments, the CO_2_ outlet flow rate was recorded by a mass flow detector (Seven Stars Hua Chuang) to ensure accuracy and was used for all subsequent efficiency calculations. And CO_2_ gas was delivered at an average rate of 11 mL min^-1^ (at room temperature and ambient pressure). The counter and reference electrodes were the Pt mesh and the Ag/AgCl (in saturated KCl solution) electrode, respectively. The ohmic loss between the working and reference electrodes was determined using electrochemical impedance spectroscopy, and potential was corrected using 85% IR compensation via the electrochemical workstation and 15% manual correction. All potentials were measured against an Ag/AgCl reference electrode and converted to the reversible hydrogen electrode (RHE) reference following the equation:

E (vs. RHE) = E (vs. Ag/AgCl)+ 0.197 + 0.0591×pH + IR

Electrochemical measurements were also performed in a flow cell (model 101017-1, Tianjin GaossUnion Co., Ltd.) using a CORRTEST CS310MA electrochemical workstation. Fumasep FAA-PK-130 anion exchange membrane (AEM) was used to separate the cathode and anode. Hg/HgO with 1 M KOH was used as reference electrode. A Ni foam was used as the anode. Each part contained 30 mL of 1 M KOH electrolyte, with flow rates of 1 mL min^-1^ for cathode chamber and 2 mL min^-1^ for anode chamber. The flow rate of CO_2_ was maintained at 15 mL min^-1^ monitored by a MODEL LF-A010 (Sichuan laifeng fluid equipment manufacturing Co., Ltd.) mass flow controller throughout the electrochemical measurements. The scan rate of the linear sweep 20 mV s^-1^. The durability test was conducted at 300 mA cm^-2^. Notably, the electrolyte was refreshed every 10 h to maintain the activity. The potentials (vs. Hg/HgO) were converted to the reversible hydrogen electrode (RHE) by the Nernst equation:

E (vs. RHE) = E (vs*.* Hg/HgO) + 0.098 V + 0.0592*pH − 0.8 × IR

Where the pH of 1 M KOH electrolyte was 13.6; The value of IR was determined by electrochemical impedance spectroscopy.

Gas phase products were routed directly into the gas sampling loop of a gas chromatograph (Agilent 8890) equipped with a thermal conductivity detector and a flame ionization detector and quantified. Liquid products were quantified using a 400 MHz 1H NMR spectrometer with water suppression.

The Faradaic efficiency (FE) of the gas products can be quantified following this equation:

$$FE=\frac{\alpha nF}{Q}=\frac{\alpha Fvn}{I\times60}$$

where α, F, v, n and I are the number of electrons transferred, Faraday constant (96485 C mol^-1^), the flow rate of gas (mL min^-1^), the number of moles for a given product and total steady-state cell current (A), respectively. The FE of the liquid products can be quantified by calibration curves of varied liquid products.^[6]^

**Cyclic voltammetry (CV) measurements.** The CV scan rate was 20 mV s^-1^ with a potential window between -1.1 V and 0.7 V vs. Ag/AgCl reference electrode. In CV curves, the region I near 0 V vs. Ag/AgCl corresponds to the oxidation of Cu^0^ to Cu^+^, and the region II was ascribed to the oxidation of Cu^+^ to Cu^2+^. Then, regions III and Ⅳ have been ascribed to the related reduction of Cu, while region V corresponds to species that are more difficult to reduce.^[3, 7, 8]^

**Double-layer capacitance (C_dl_).** Electrochemically active surface area (ECSA) is a key parameter to assess the intrinsic activity of electrocatalysts.^[3, 9, 10]^ Here, we opted to assess the ECSA by capacitance measurement because of Cu NWs coated with a porous AlOx shell. In our case the material remains conductive and the plotted total current density against scan rate leads to a straight line, which confirms that the capacitive contribution mainly comes from the double layer charging.^[3, 11]^ CV analysis was performed at the range of 50 mV around the open circuit potential (vs*.* Ag/AgCl). the electrochemically active surface area (ECSA) of the electrode can be calculated based on the following equation:

E$CSA=Rf*S$

where S is the geometric area of the electrode (cm^2^) and R_f_ is roughness factor.

$$Rf=Cdl/a$$

where a is 29 μF cm^-2^ for Cu, respectively.

**In-situ Raman spectroscopy.** Raman measurements were performed using a laser confocal Raman spectrometer (LabRAM, Horiba-JY) in a three-electrode electrochemical cell. The working electrode was prepared by using Cu NWs or Cu@AlOx_n=0.5_ NWs loaded on hydrophobic carbon paper (1.5×1.5 cm^2^, 0.8 mg cm^-2^). A carbon rod and Ag/AgCl (saturated KCl) acted as the counter electrode and reference electrode, respectively. 0.1 M KHCO_3_ aqueous solution was used as electrolyte. Raman spectra were collected in the range of 100 to 3500 cm^−1^ using 785 nm laser.

**Computational details.** All of the calculations are performed in the framework of the density functional theory with the projector augmented plane-wave method, as implemented in the Vienna ab initio simulation package (VASP).^[12, 13]^ The generalized gradient approximation (GGA) proposed by Perdew, Burke, and Ernzerhof (PBE) is selected for the exchange-correlation potential.^[14, 15]^ The long-range van der Waals interaction is described by the Density Functional Theory (DFT)-D3 approach.^[16]^ The cut-off energy for plane wave is set to 480 eV. The energy criterion is set to 10^-5^ eV in iterative solution of the Kohn-Sham equation. A 2×2×1 k-point mash in Brillouin zone was applied in this case. The system was equilibrated and analyzed in the NVT ensemble (constant particle number, volume, and temperature) at a temperature of 298.15 K. A time step of 2 fs was employed to ensure accurate integration of the equations of motion. All the structures are relaxed until the residual forces on the atoms have declined to less than 0.02 eV/Å. Data analysis and visualization are carried out with the help of VASPKIT code and VESTA. ^[17, 18]^ To avoid interlaminar interactions, a vacuum spacing of 20 Å is applied perpendicular to the slab.

Here, differences in Gibbs free energy (ΔG) for intermediates defined as:

|  | $\Delta G=\Delta E+\Delta E_{\mathrm{ZPE}}-T\Delta S+\Delta G_{U}$ |  |
| --- | --- | --- |

where ΔG is the total energy difference between the slab and respective terminations computed by DFT-PBE. ΔEZPE and TΔS denotes differences in zero-point energy and entropy between adsorbed states of reaction intermediates and gap phase, respectively. T is the room temperature (298.15 K). ΔGU = −eU, whereby U is the electrode potential.

The adsorption energy Eads is expressed as

|  | $\Delta E\text{ads}=E_{\text{A+B}}-E_{A}-E_{B}$ |
| --- | --- |

where $E_{A+B}$ is the total energy of slab A model with B adsorption, $E_{A}$ is the energy of a A slab, and $E_{B}$ is that for a B molecule.


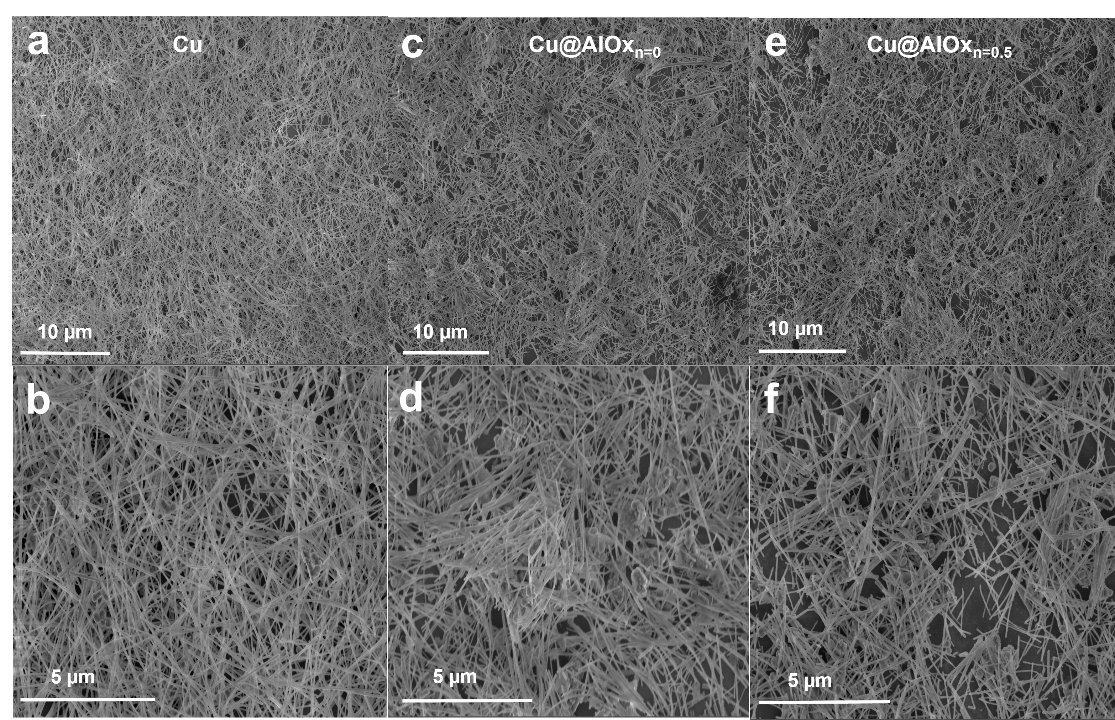


Figure S1. SEM imaging for as-synthesized (a,b) Cu NWs, (c,d) Cu@AlOx_n=0_ NWs and (e,f) Cu@AlOx_n=0.5_ NWs.


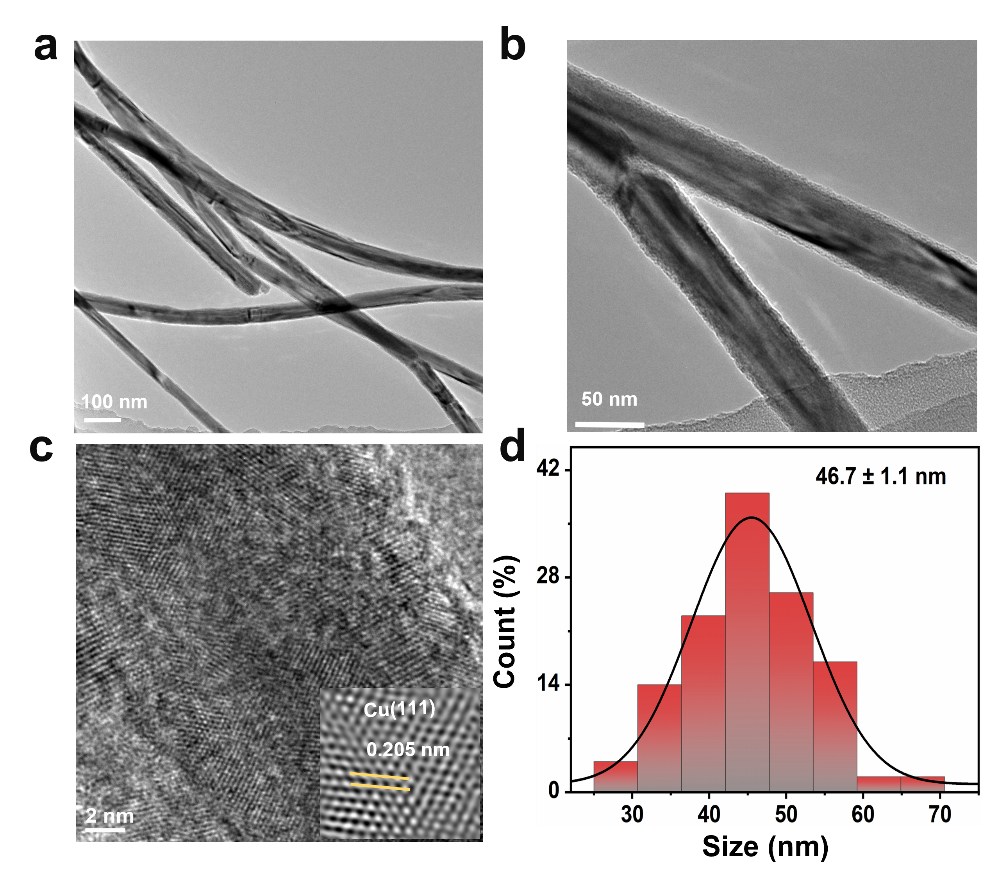


Figure S2. (a,b) TEM and (c) HRTEM imaging, and (d) size distribution of as-synthesized Cu NWs.


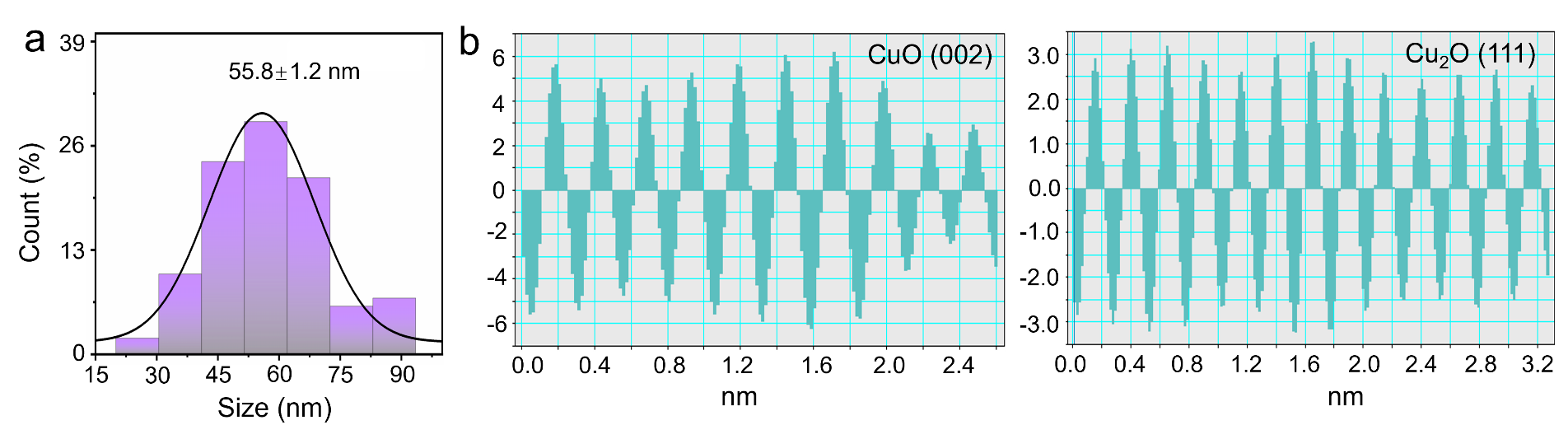
Figure S3. (a) Size distribution of as-synthesized Cu@AlOx_n=0.5_ NWs. (b) The intensity profiles of the lattice spacing shown in the marked area in Figure 1c.


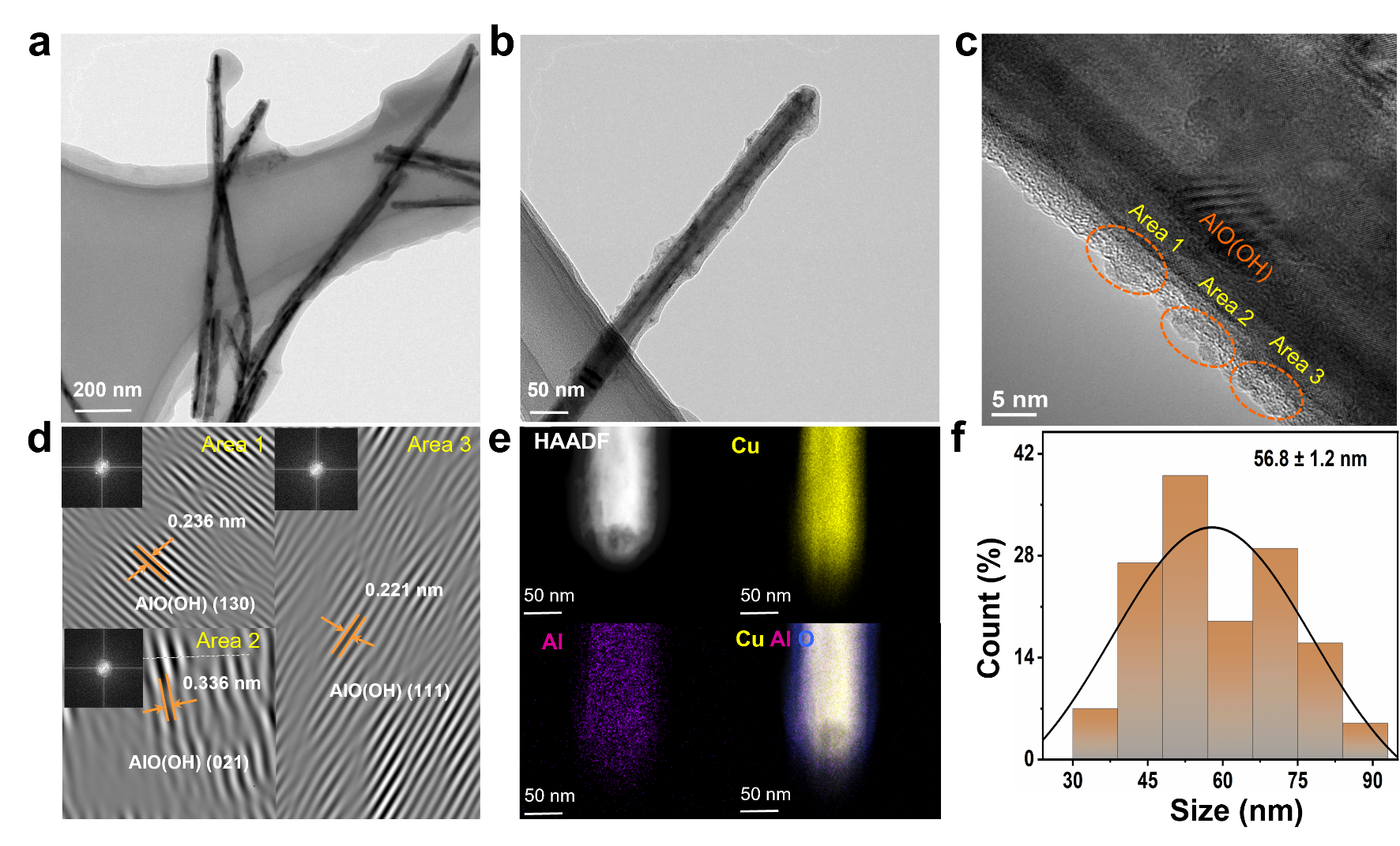


Figure S4. (a,b) TEM and (c) HRTEM imaging with (d) corresponding fast Fourier transform (FFT) and inverse FFT patterns. (e) HAADF mapping and (f) size distribution of as-synthesized Cu@AlOx_n=0_ NWs.


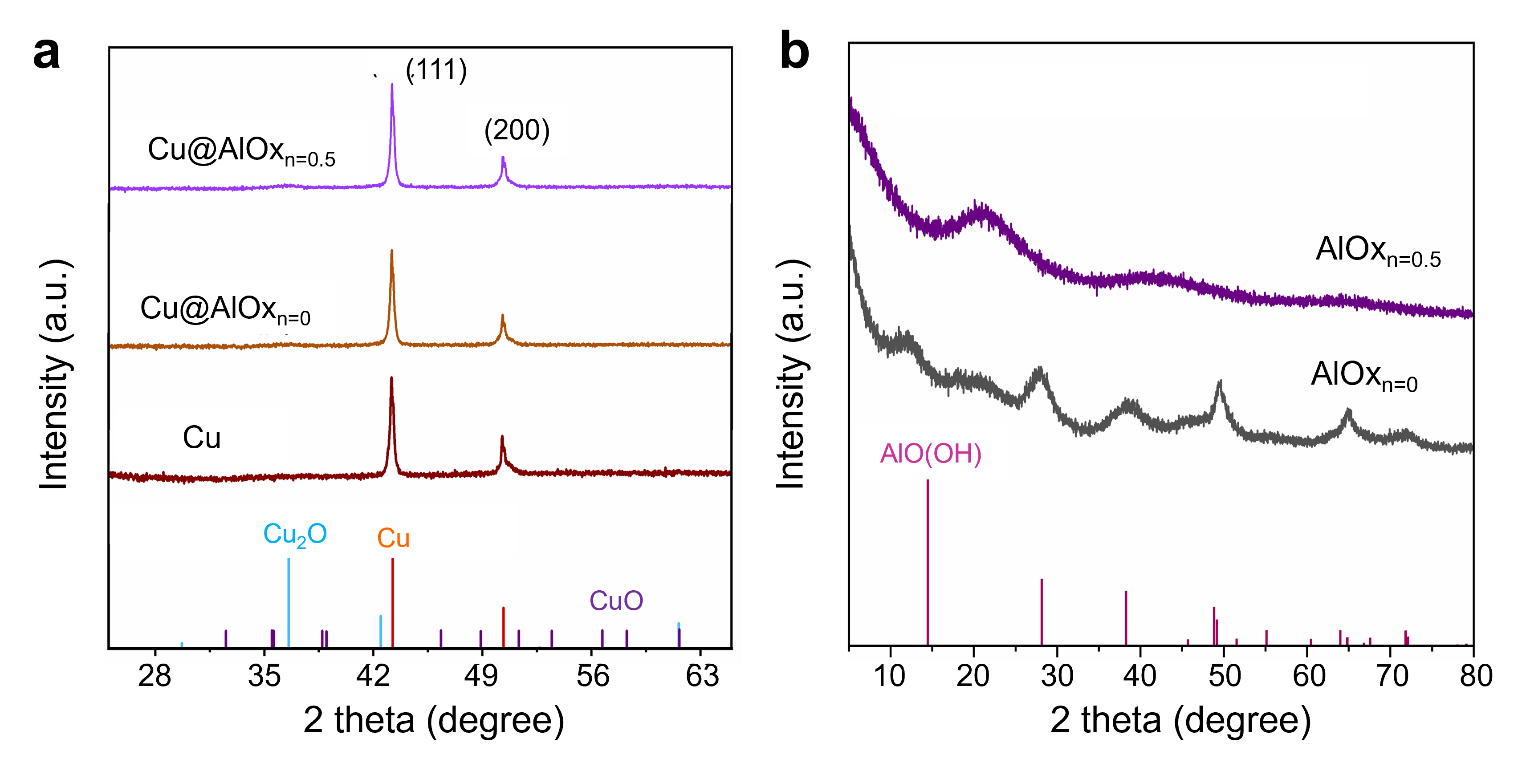


Figure S5. (a) XRD patterns of Cu NWs and different Cu@AlOx NWs. The PDF standard cards are PDF#70-3038-Cu, PDF#77-0199-Cu_2_O and PDF#80-0076-CuO. (b) XRD patterns of different AlOx samples, and PDF standard cards is PDF#83-1506-AlO(OH)-Boehmite.


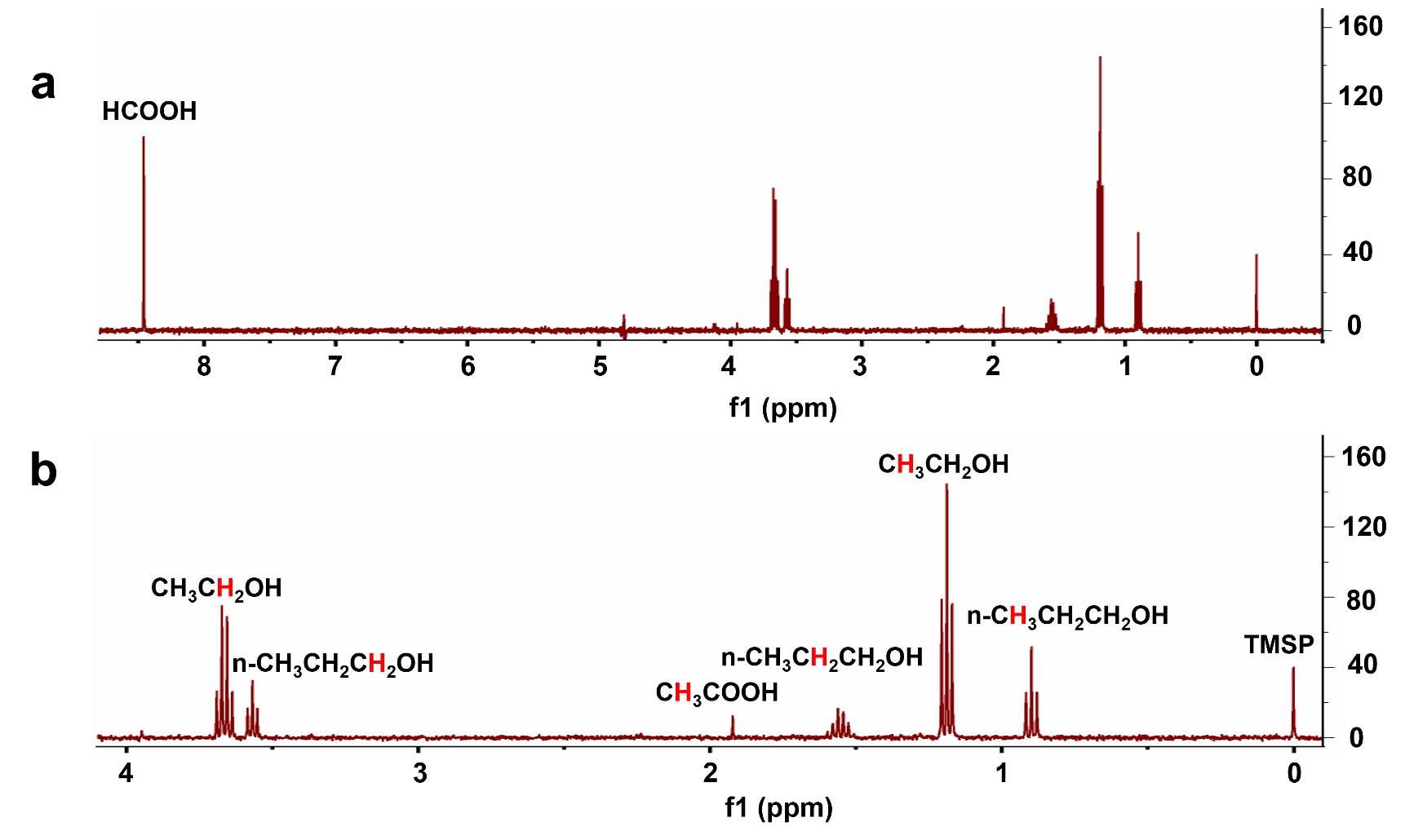


Figure S6. (a) ^1^H NMR spectroscopy for Cu@AlOx_n=0.5_ NWs operating at -1.1 V vs. RHE for 16 h in an H-cell reactor, and (b) the magnified spectrum details.


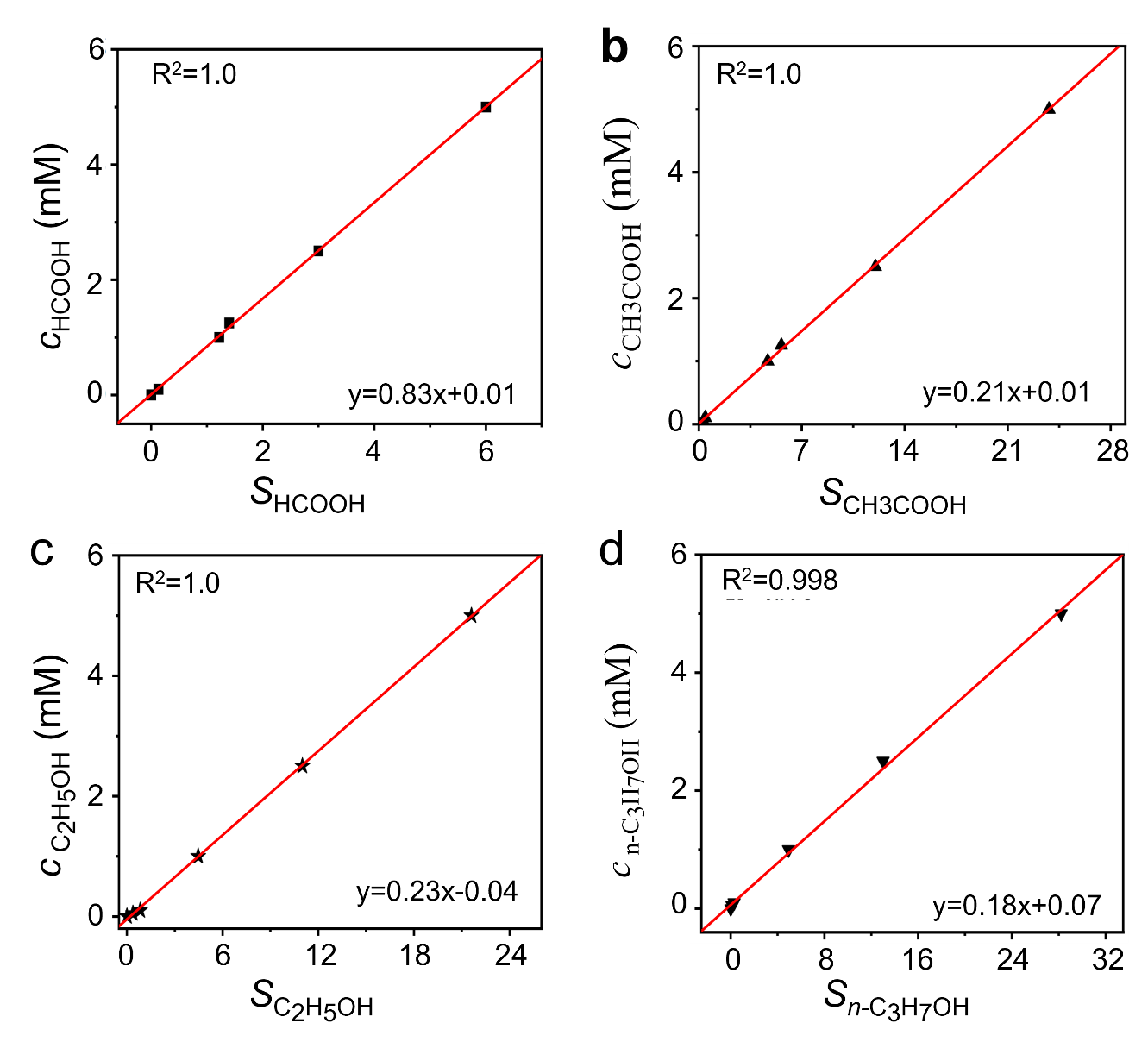


Figure S7. Calibration curves of liquid products: (a) HCOOH, (b) CH_3_COOH, (c) C_2_H_5_OH, and (d)
n-C_3_H_7_OH.


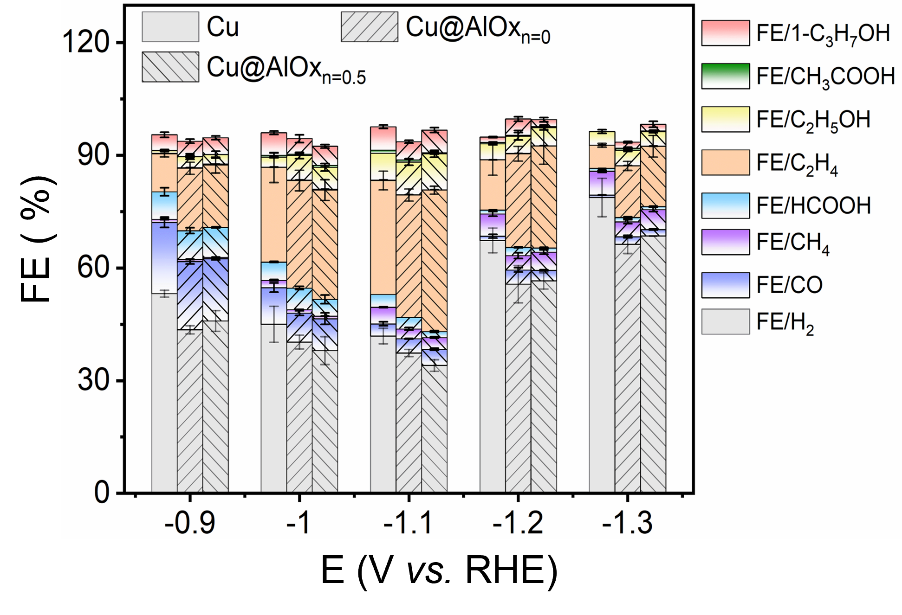


Figure S8. Comparison of the eCO_2_RR performance between pristine Cu NWs and different Cu@AlOx NWs in 0.1M KHCO_3_ at different potentials in an H-cell configuration.


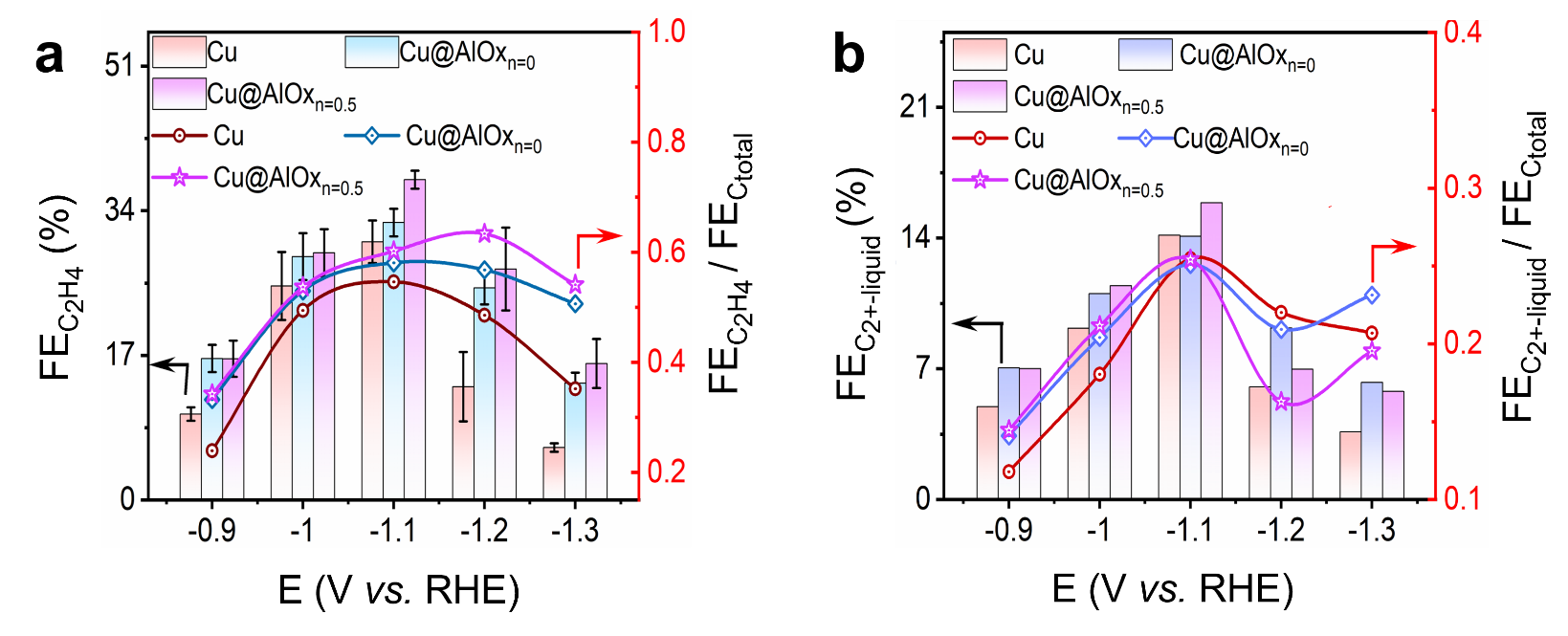


Figure S9. The comparison of FE toward (a) C_2_H_4_ and (b) C_2+_ liquid product between pristine Cu NWs and different Cu@AlOx NWs at a series of potentials. The right-hand axis represents the percentage of (a) C_2_H_4_ or (b) C_2+_ liquid product in total C production. All data are obtained via an H-cell configuration.


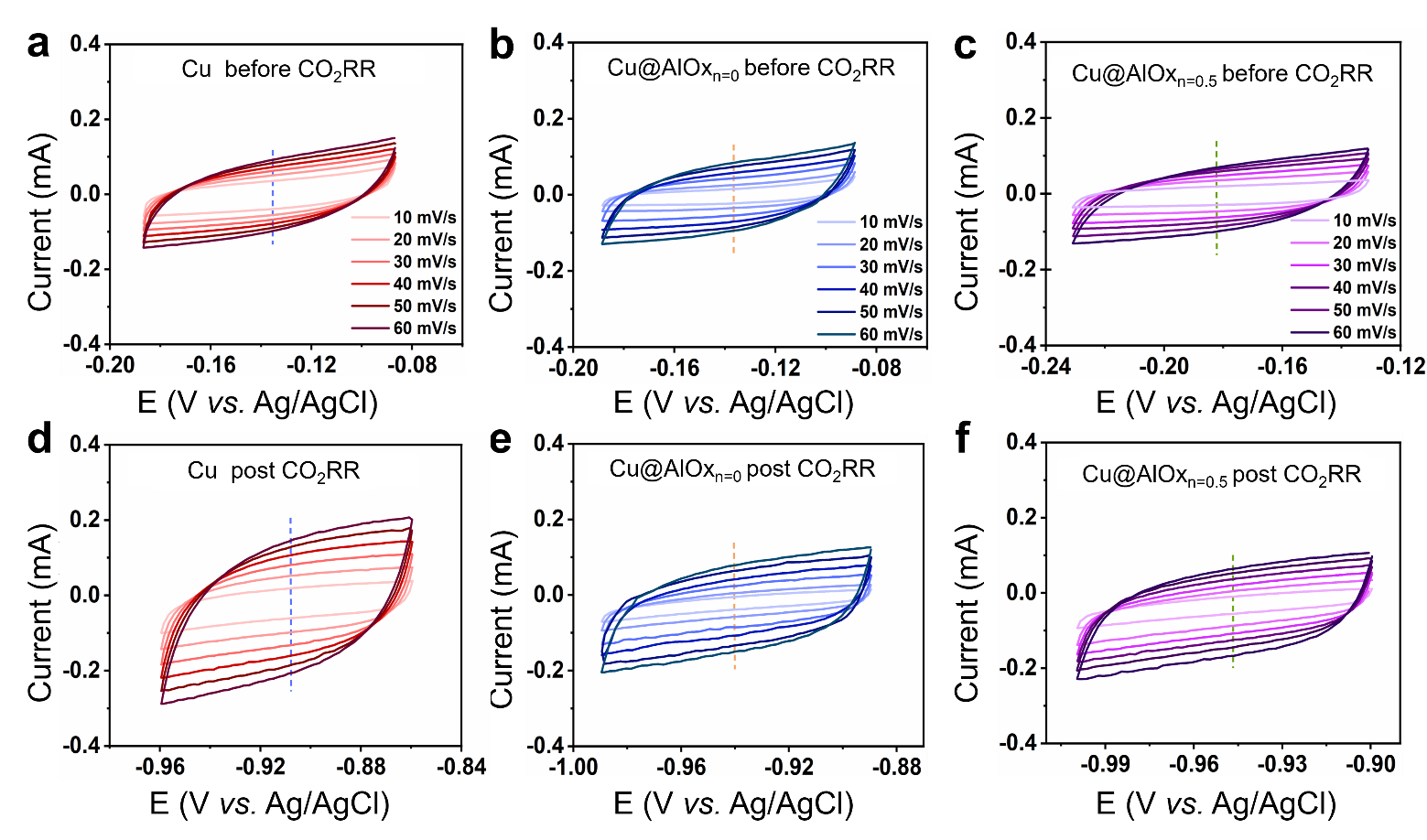


Figure S10. Measurements of the *C*_dl_ by cyclic voltammetry curves with different scan rates for Cu NWs, Cu@AlOx_n=0_ NWs, and Cu@AlOx_n=0.5_ NWs (a,b,c) before and (d,e,f) after 48 h operation at scan rates ranging from 10 to 60 mV s^-1^.


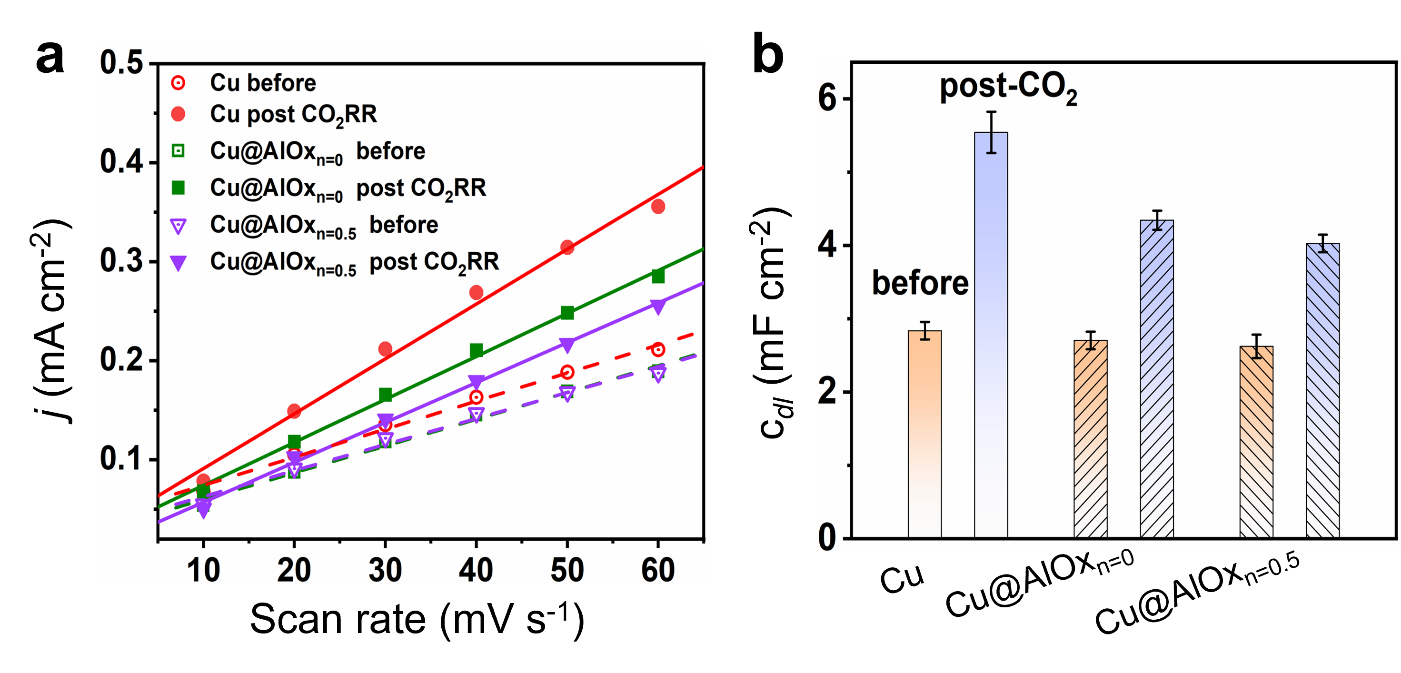


Figure S11. (a) Measurements of the *C*_dl_ of Cu NWs and different Cu@AlOx NWs correlating the capacitive current density with the CV scan rate; (b) the determination of *C*_dl_ before and after 48 h of eCO_2_RR measurement.


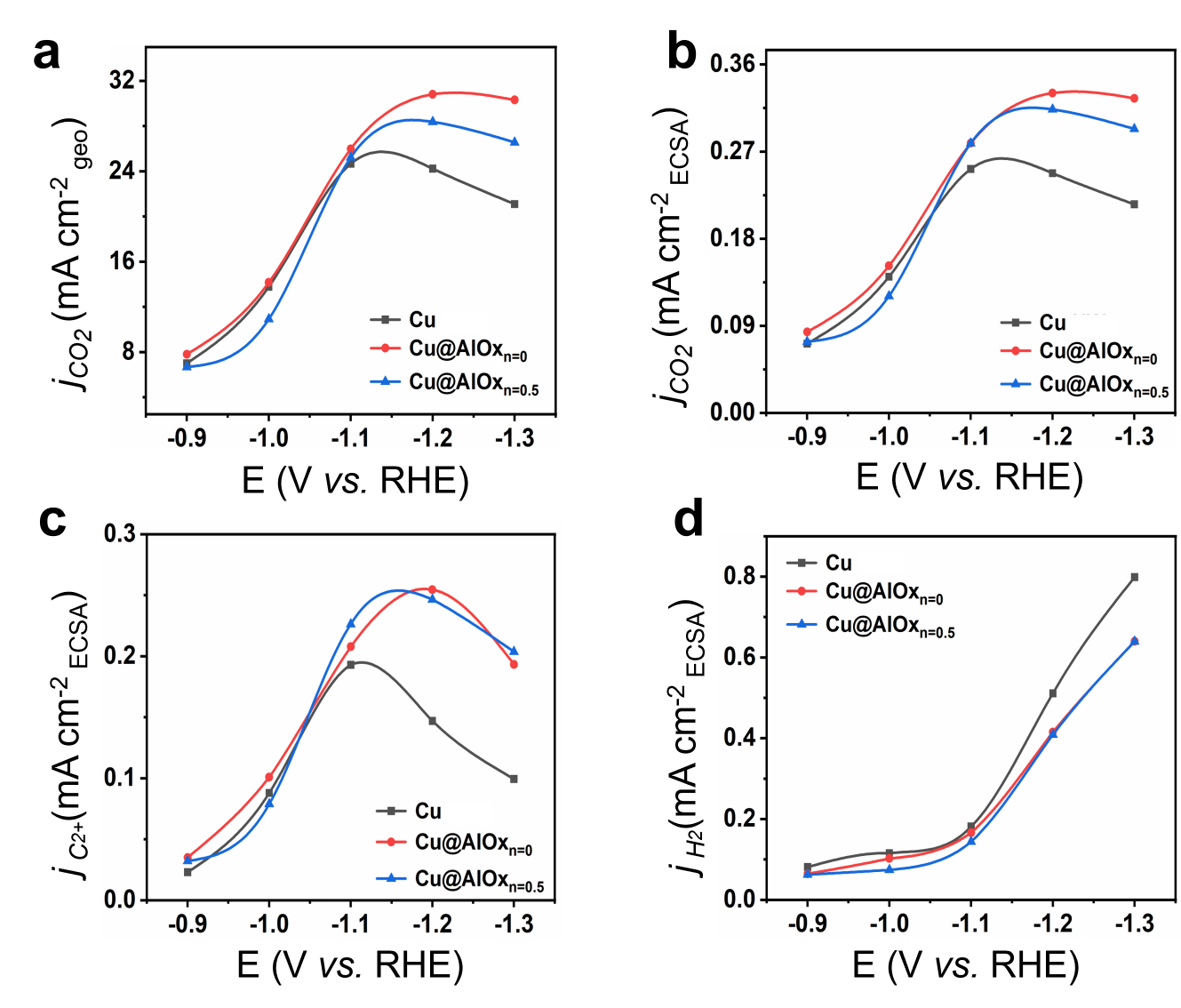


Figure S12. (a,b) The partial CO_2_-reudcing current density normalized by geometric area and ECSA for pristine Cu NWs and different Cu@AlOx NWs, respectively. ECSA-normalized partial current density of (c) C_2+_ products and (d) H_2_ for pristine Cu NWs and Cu@AlOx NWs.


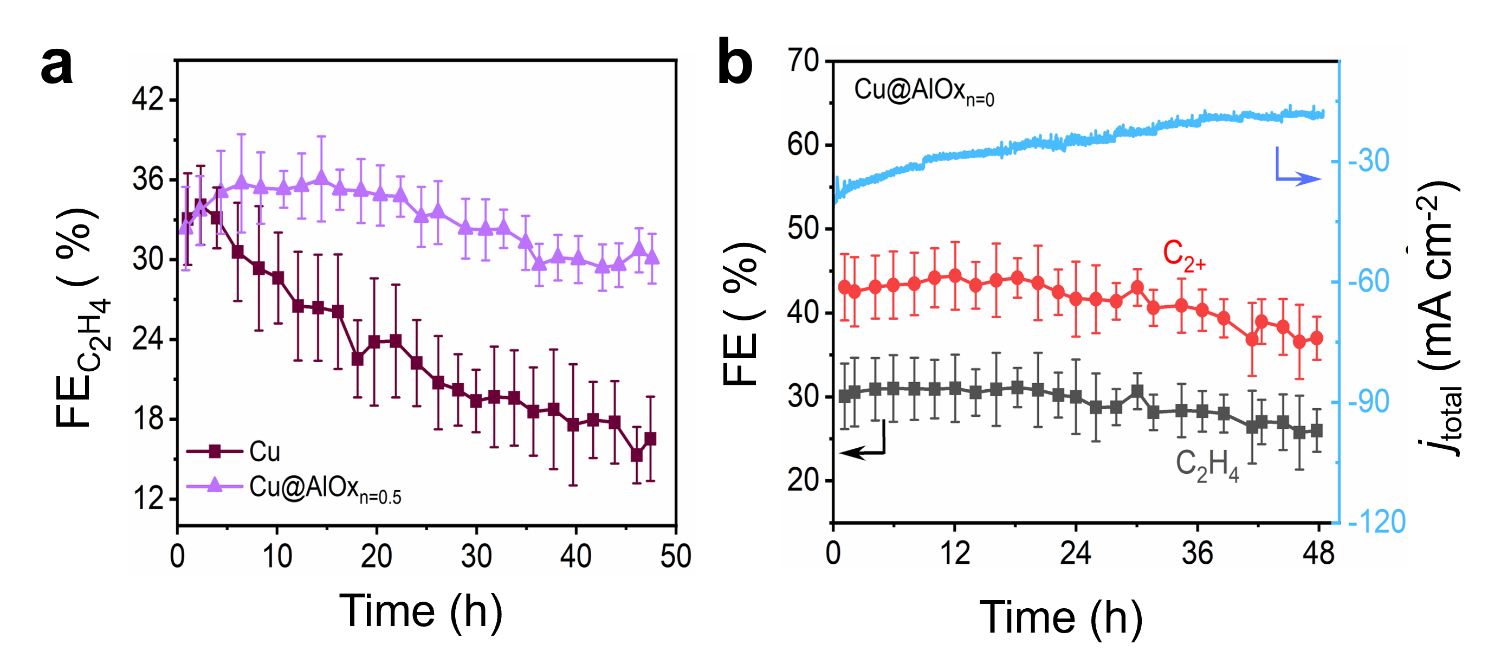


Figure S13. (a) The stability test of C_2_H_4_ for pristine Cu NWs and Cu@AlOx_n=0.5_ NWs at -1.1 V vs*.* RHE in 0.1 M CO_2_-saturated KHCO_3_. (b) Long-term stability test of C_2_H_4_ and C_2+_ product for Cu@AlOx_n=0_ NWs. The left-hand axis is the FE of various C products, while the right-hand axis is the total current density. All measurements were performed via an H-cell configuration.


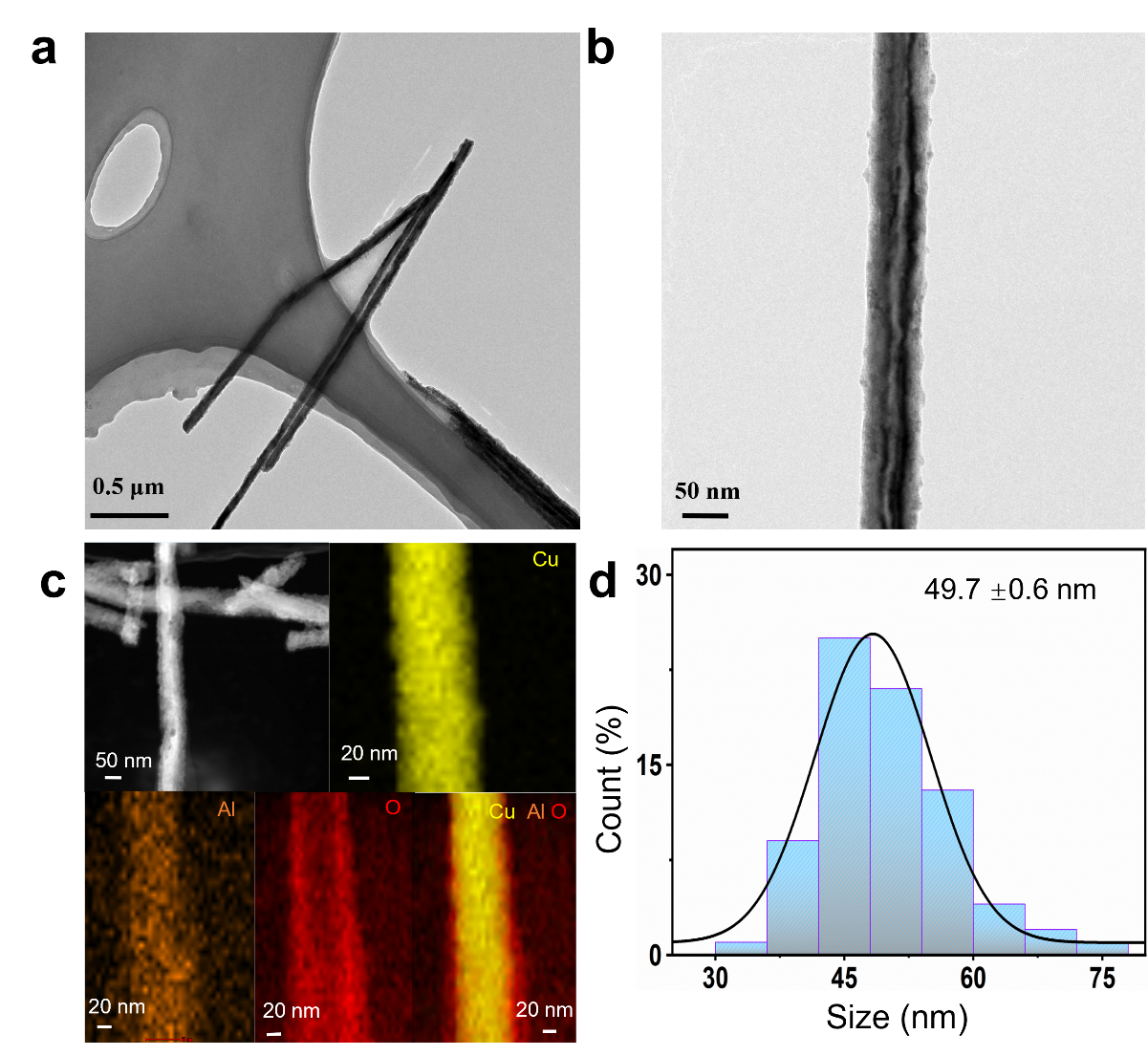


Figure S14. (a,b) TEM imaging of thin-Cu@AlOx_n=0.5_ NWs. (c) Representative HAADF-STEM imaging and corresponding EDS elemental maps of Cu, Al, O and their overlap and (d) size distribution of thin-Cu@AlOx_n=0.5_ NWs.


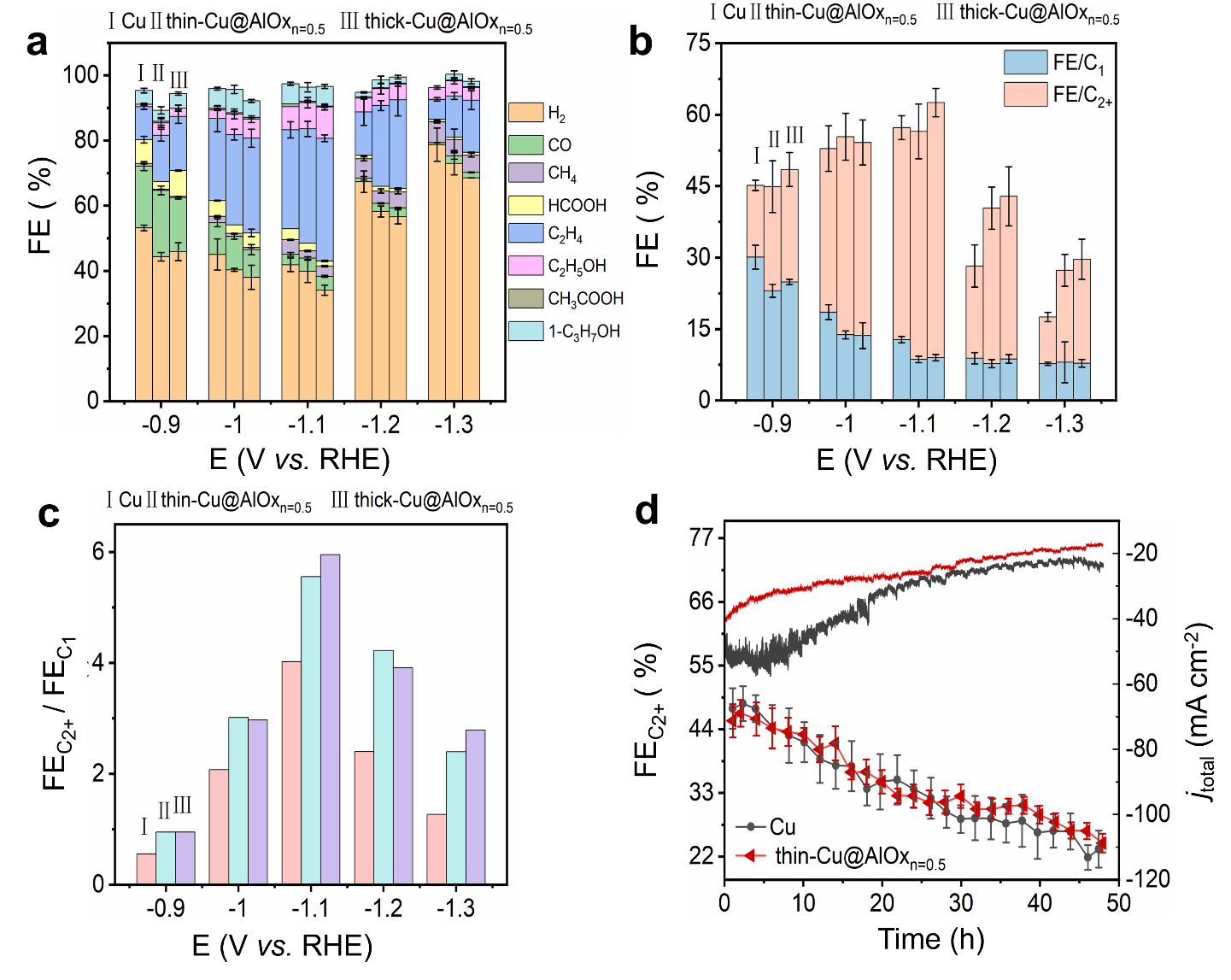


Figure S15. (a) Comparison of the eCO_2_RR performance between pristine Cu NWs, Cu@AlOx_n=0.5_ NWs, and thin-Cu@AlOx_n=0.5_ NWs in 0.1 M KHCO_3_ at different potentials in an H-cell reactor. (b) FE of C_1_ and C_2+_ products for the three catalysts at various potentials. (c) The ratios of C_2+_/C_1_ products as a function of the applied potential, illustrating the enhanced C_2+_ selectivity for Cu@AlOx_n=0.5_ NWs and thin-Cu@AlOx_n=0.5_ NWs. (d) Comparison of long-term stability test of C_2+_ production between pristine Cu NWs and thin-Cu@AlOx_n=0.5_ NWs.


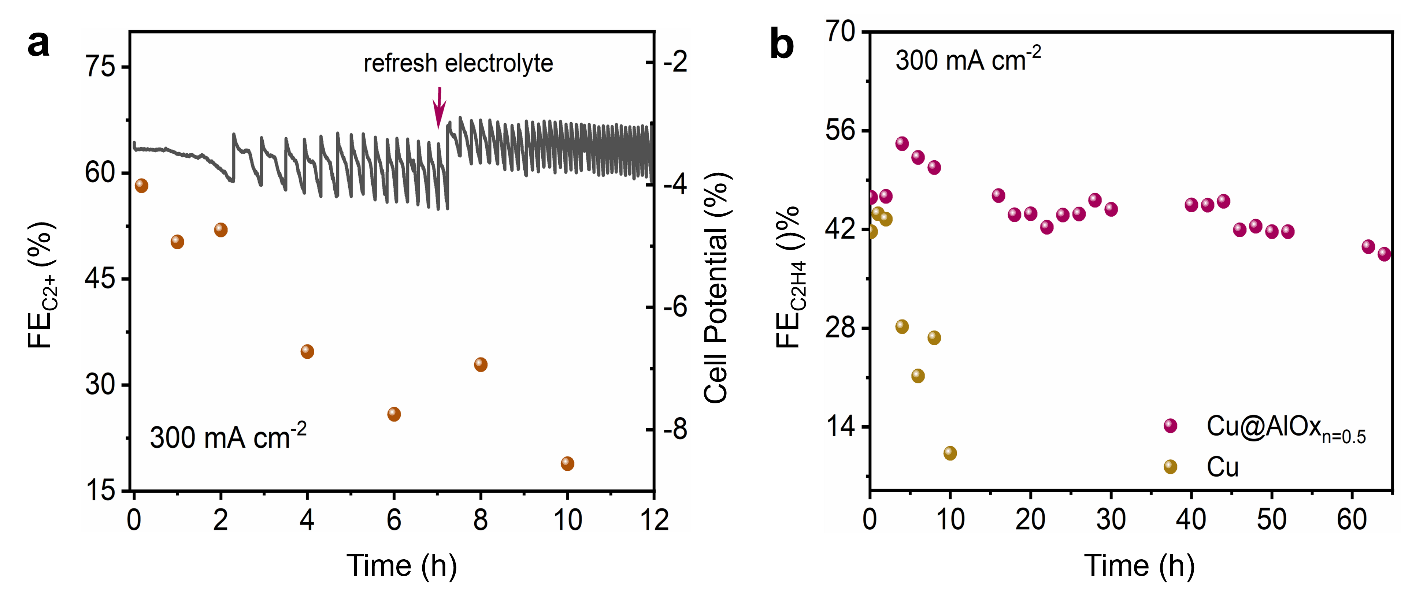


Figure S16. (a) Stability tests for C_2+_ production on pristine Cu NWs at 300 mA cm^-2^ in a flow cell reactor. (b) Stability tests for C_2_H_4_ production on pristine Cu NWs (10 h) and Cu@AlOx_n=0.5_ NWs (64 h) during eCO_2_RR operation in a flow cell reactor.


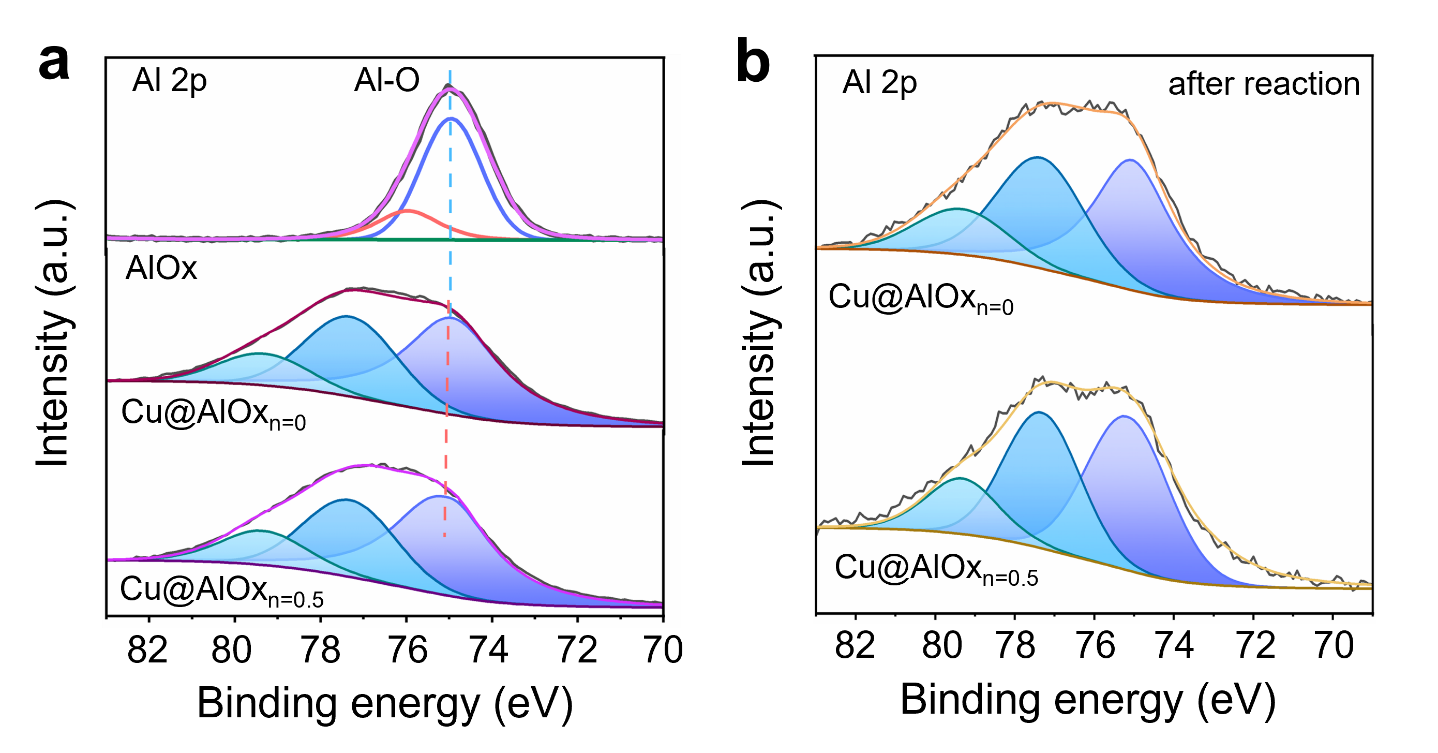


Figure S17. (a) High-resolution XPS spectra of Al 2p for AlOx reference, Cu@AlOx_n=0_ NWs, and Cu@AlOx_n=0.5_ NWs before eCO_2_RR measurements. (b) High-resolution XPS spectra of Al 2p for Cu@AlOx_n=0_ NWs and Cu@AlOx_n=0.5_ NWs after 24 h of eCO_2_RR measurements.


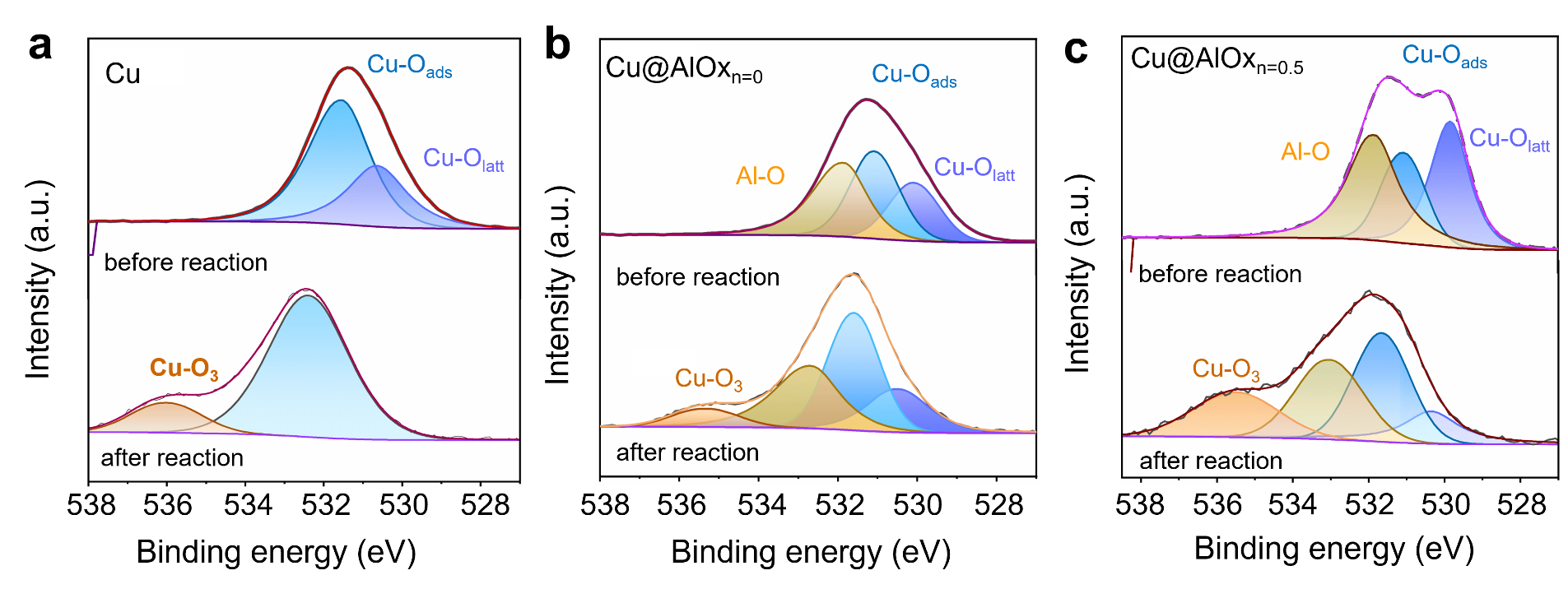


Figure S18. High-resolution XPS spectra of O 1s for (a) Cu NWs, (b) Cu@AlOx_n=0_ NWs, and (c) Cu@AlOx_n=0.5_ NWs before and after 24 h of eCO_2_RR measurements. In the spectra, the peaks include lattice oxygen (Cu-O_latt_), adsorb oxygen (Cu-O_ads_), and weakly bounded oxygen species (Cu-O_3_: CO_3_^2-^, H_2_O, and OH), respectively.


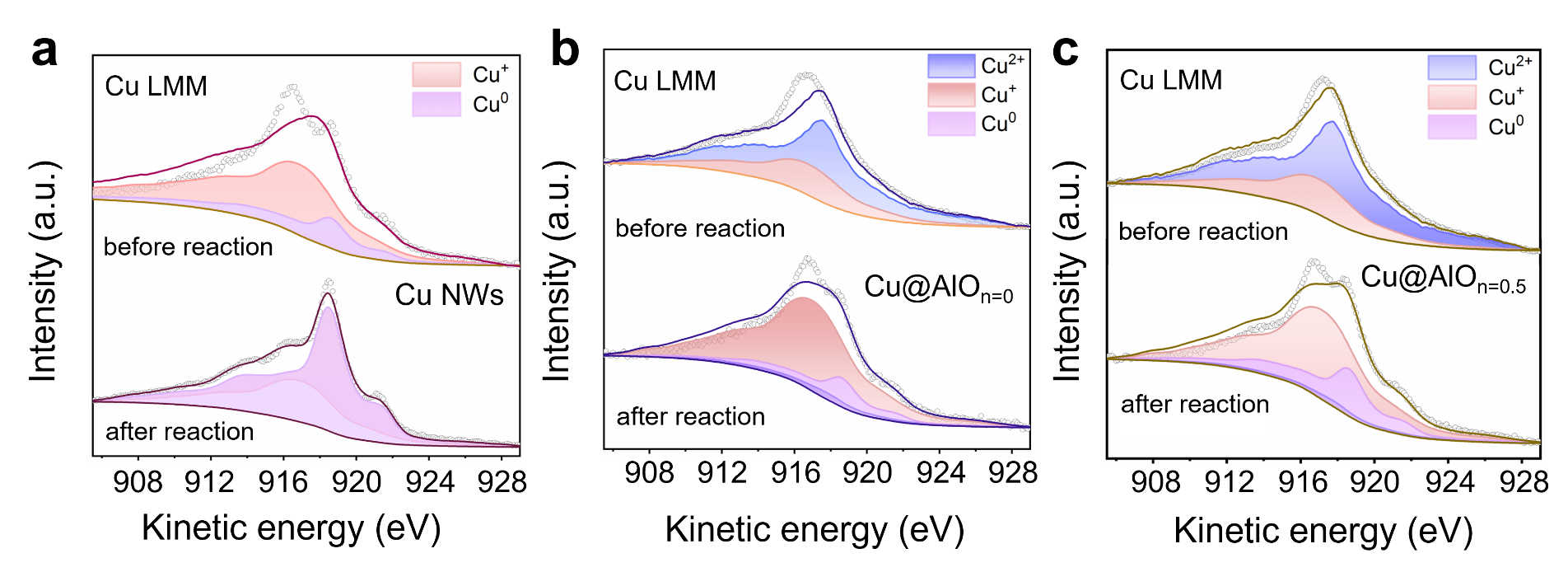


Figure S19. The Cu LMM for (a) pristine Cu NWs, (b) Cu@AlOx_n=0_ NWs, and (c) Cu@AlOx_n=0.5_ NWs before and after 24 h of eCO_2_RR measurements, respectively.


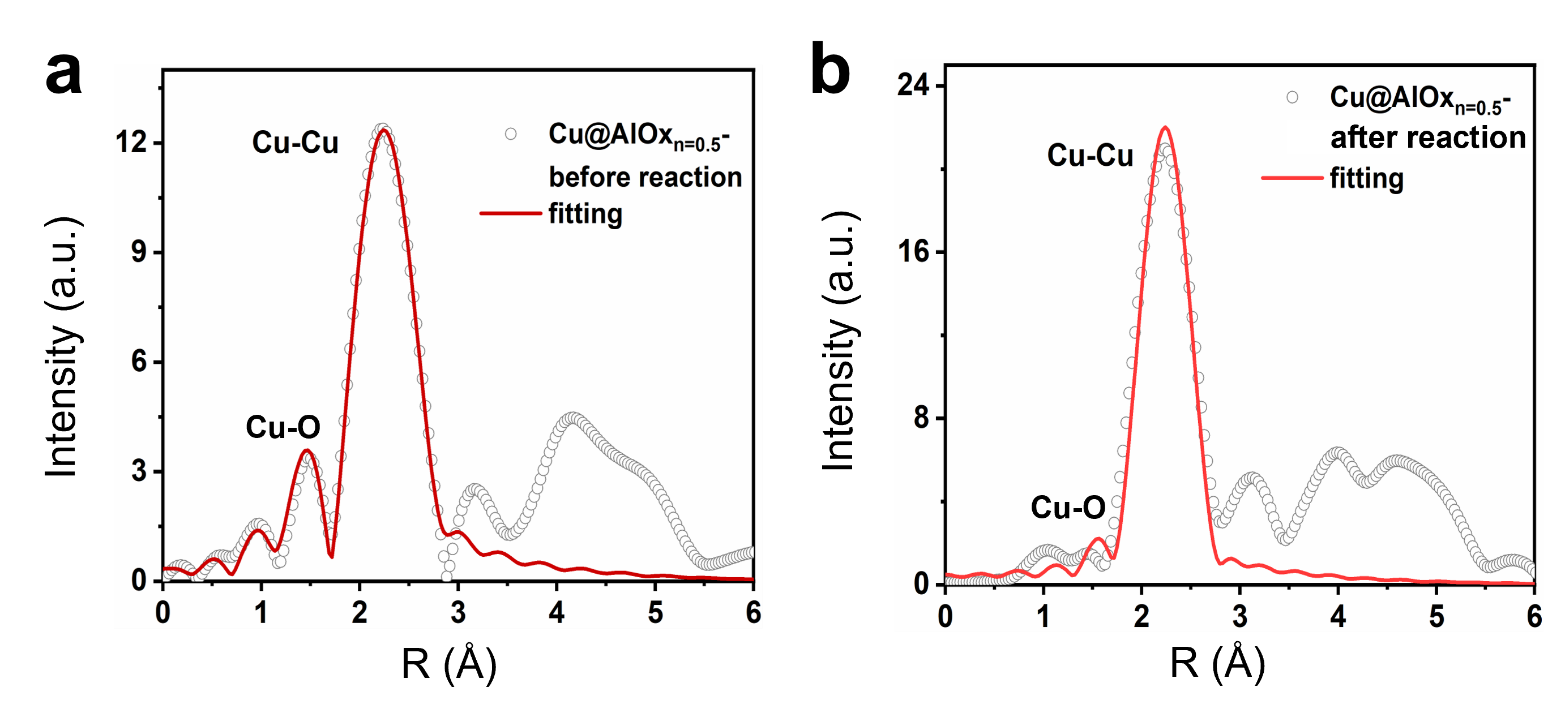


Figure S20. Fourier-transformed Cu FT(k^3^)-EXAFS spectrum of Cu@AlOx_n=0.5_ NWs (a) before and (b) after 24 h of eCO_2_RR measurements at -1.1 V *vs.* RHE and its fitting result.


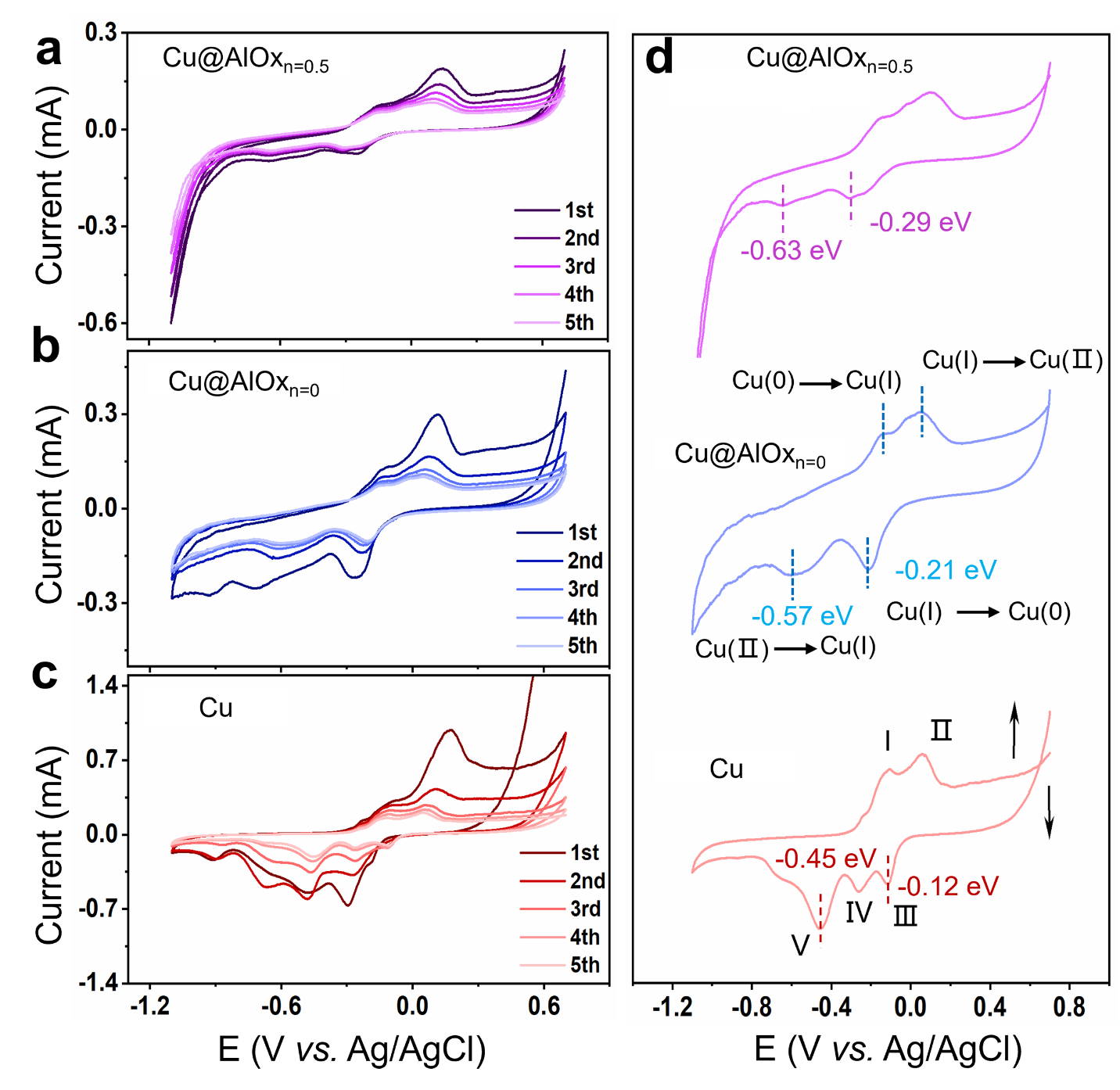


Figure S21. (a,b,c) Successive cycles of CV measurements from cathodic to anodic wave, run in 0.1M KHCO_3_ at 20 mV s^-1^ scan rate for Cu@AlOx_n=0.5_ NWs (violet, a), Cu@AlOx_n=0_ NWs (blue, b) and pristine Cu NWs (red, c). (d) CV curves from cathodic to anodic wave for Cu@AlOx_n=0.5_ NWs (violet), Cu@AlOx_n=0_ NWs (blue), pristine Cu NWs (red).


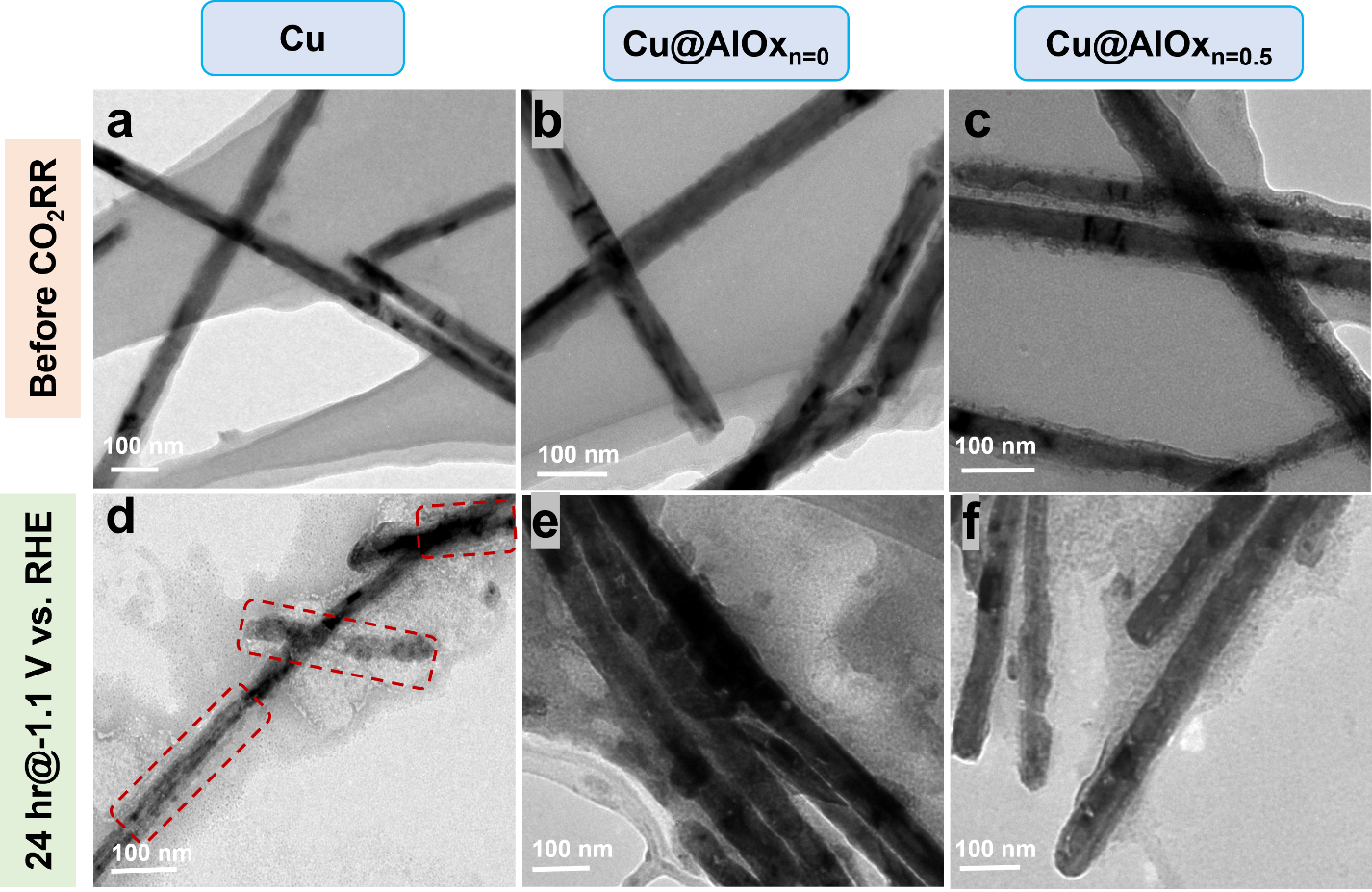


Figure S22. TEM imaging of (a,d) pristine Cu NWs, (b,e) Cu@AlOx_n=0_ NWs, and (c,f) Cu@AlOx_n=0.5_ NWs (a,b,c) before and (d,e,f) after 24 h of eCO_2_RR operation.


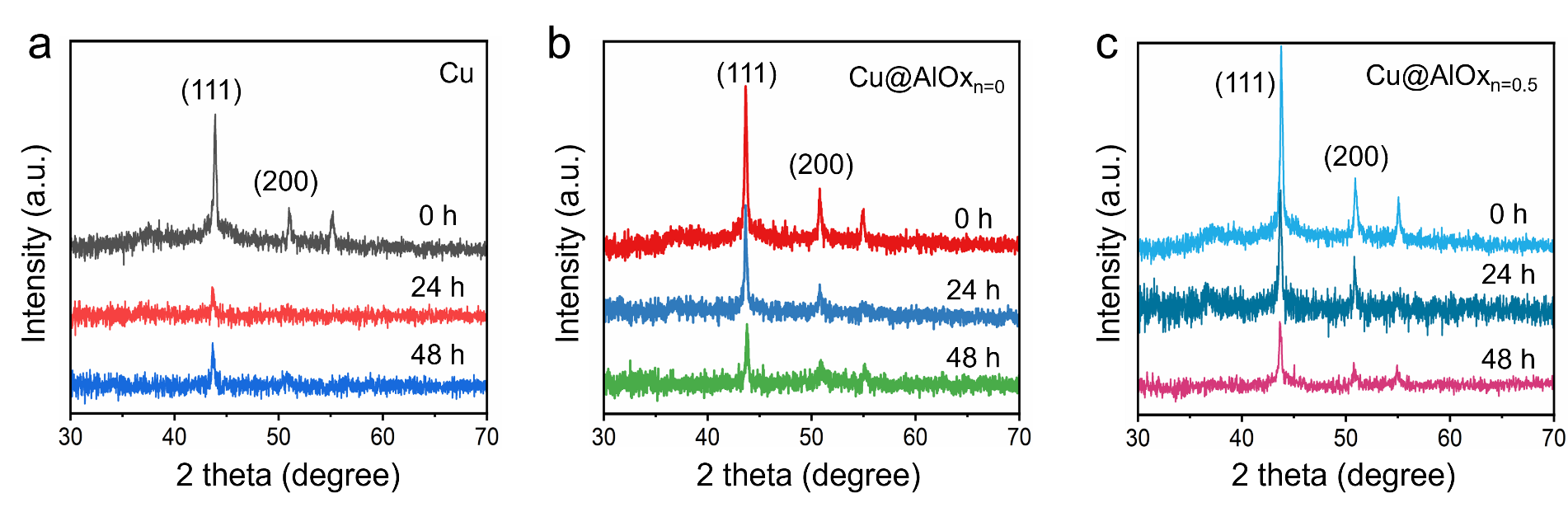


Figure S23. XRD patterns of (a) pristine Cu NWs, (b) Cu@AlOx_n=0_ NWs, and (c) Cu@AlOx_n=0.5_ NWs before, after 24 h and after 48 h of eCO_2_RR measurements.


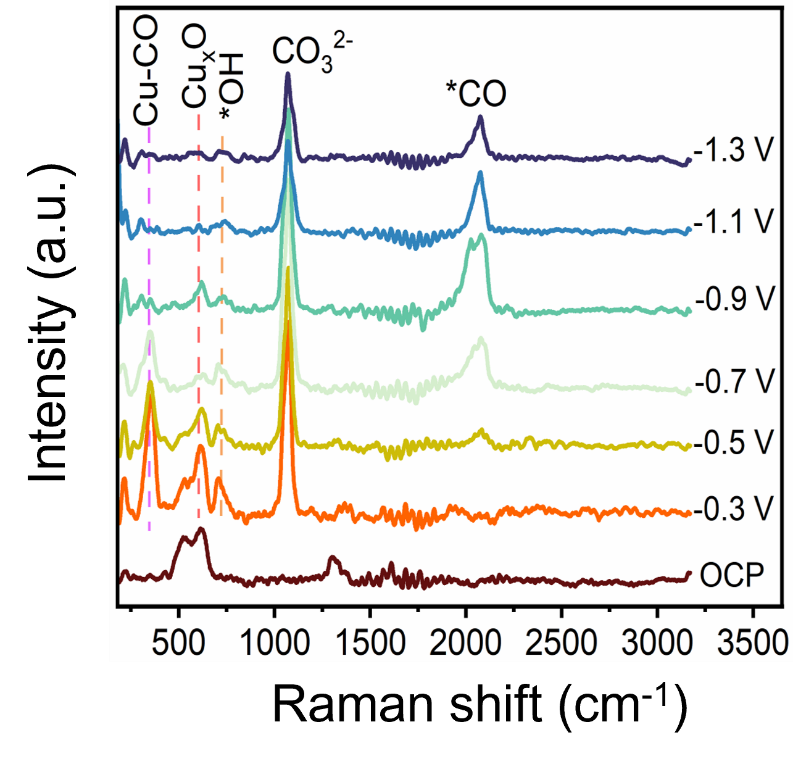


Figure S24. In situ Raman spectra of Cu@AlOx_n=0.5_ NWs at different applied potentials from OCP to -1.3 V *vs.* RHE, with a full wavenumber range from 200 to 3200 cm^-1^.


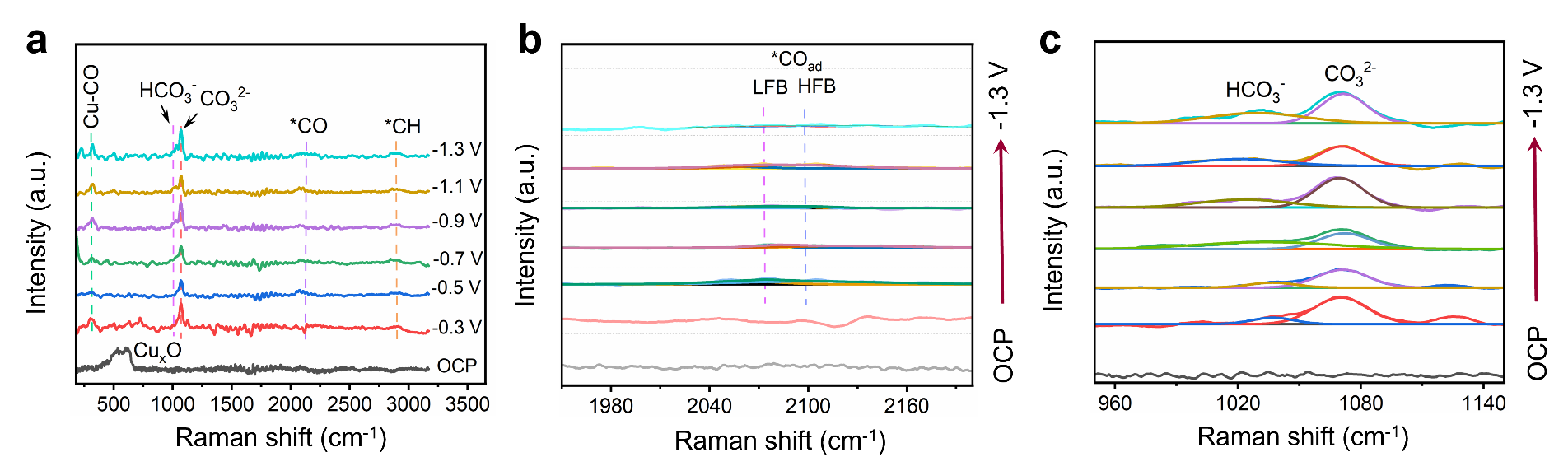


Figure S25. (a) In situ Raman spectra of pristine Cu NWs at different applied potentials from OCP to -1.3 V *vs.* RHE, with a full wavenumber range from 200 to 3200 cm^-1^. (b) The spectra of *CO_ad_ and (c) HCO_3_^-^/CO_3_^2-^ adsorption of pristine Cu NWs at different applied potentials from OCP to -1.3 V *vs.* RHE.


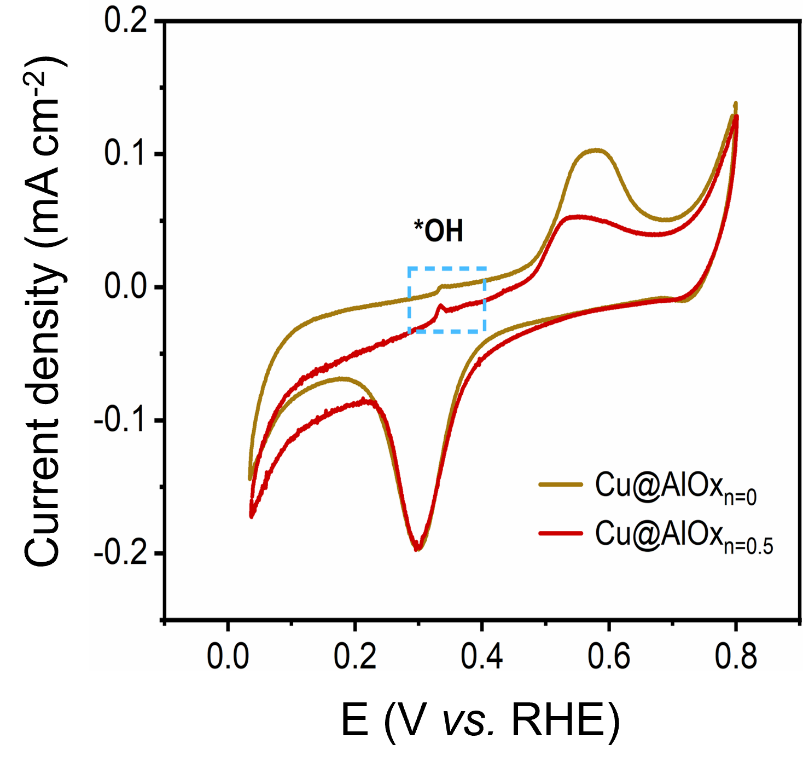


Figure S26. CV curves for Cu@AlOx_n=0_ NWs and Cu@AlOx_n=0.5_ NWs at a scan rate of 20 mV s^-1^ with 0.1 M KOH electrolyte. The peak with *OH represents the adsorption of *OH.


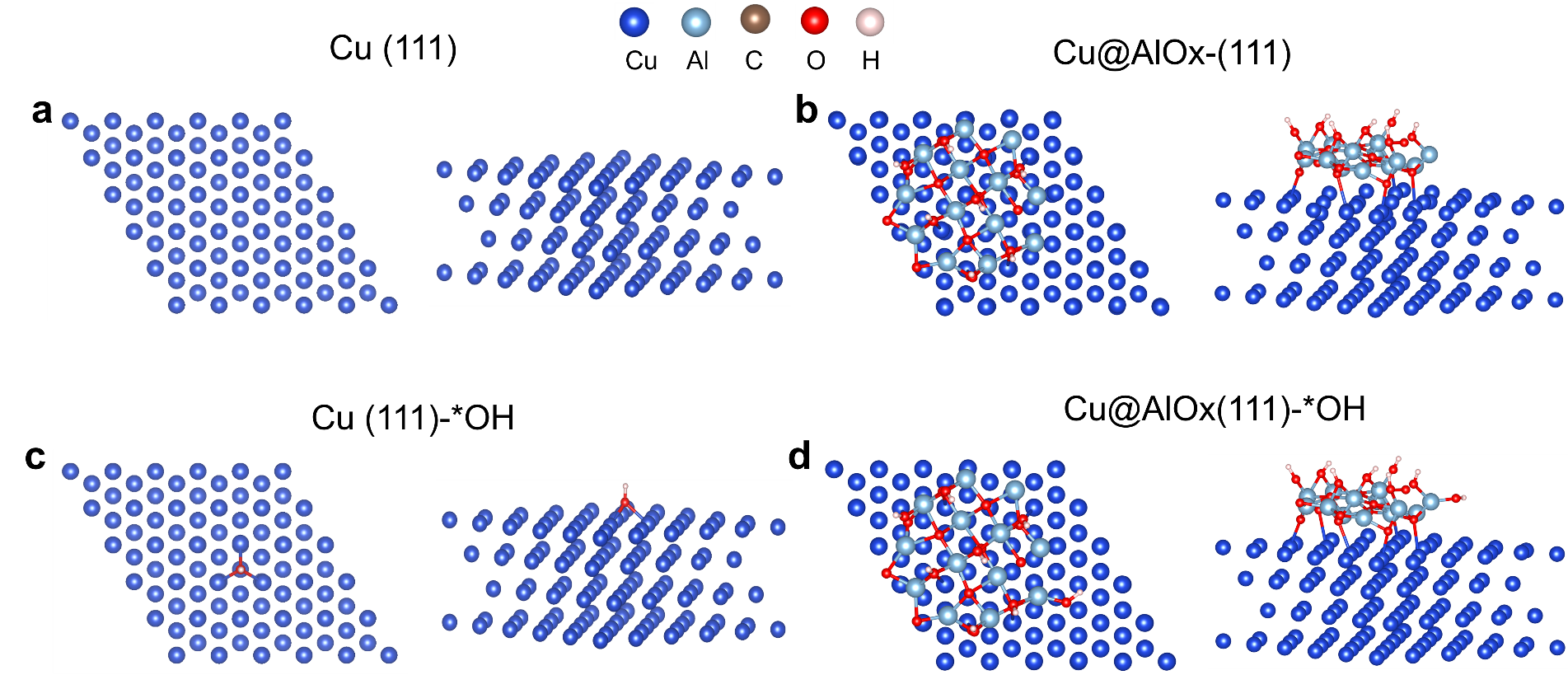


Figure S27. Top and side view of DFT models for (a) pristine Cu and (b) Cu@AlOx. The top and side view of *OH adsorption on (c) pristine Cu and (d) Cu@AlOx. The Cu (111) facet is used in all models.


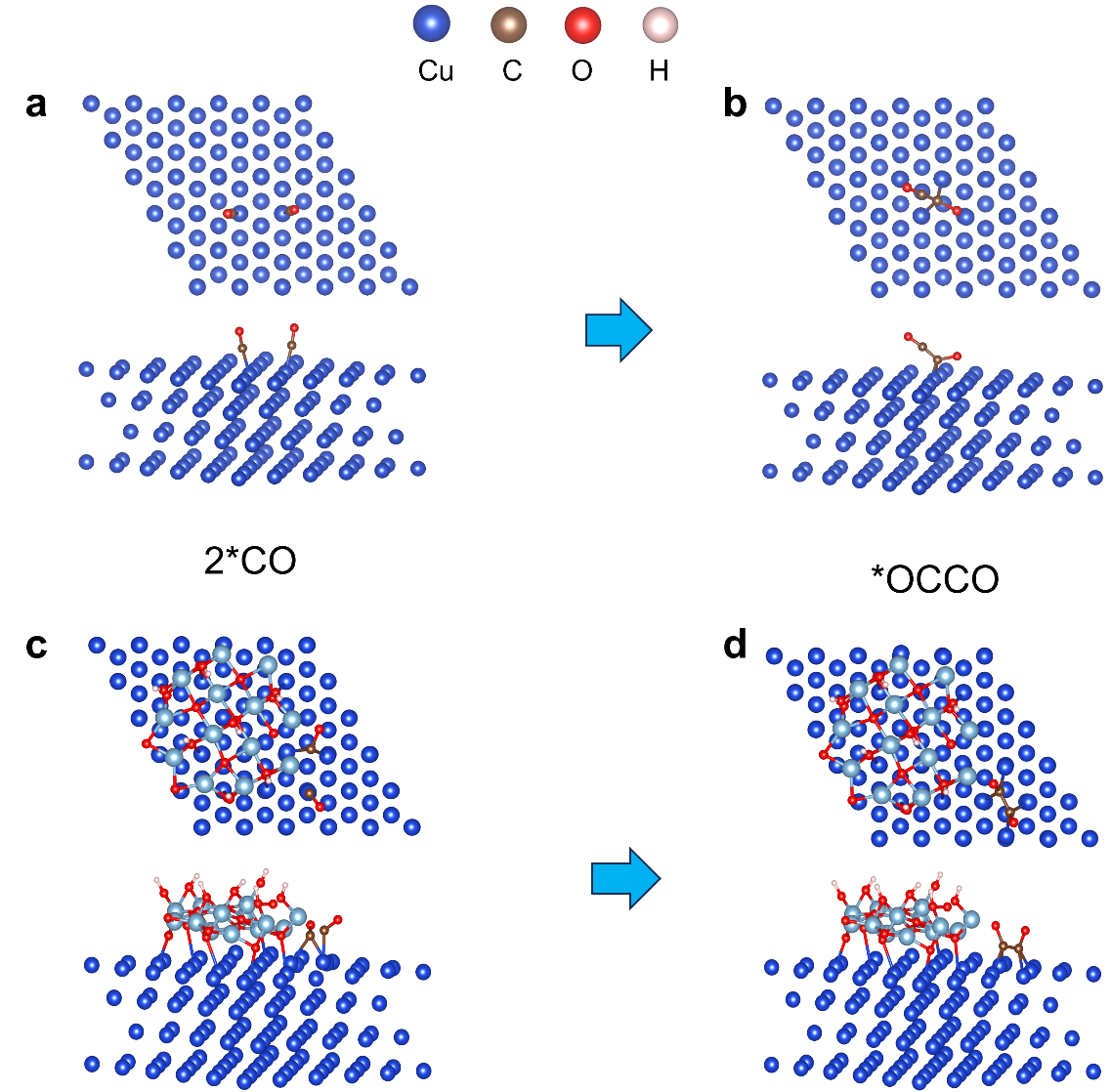


Figure S28. Top and side views of the DFT models showing dimerization of *CO to *COCO on (a,b) pristine Cu and (c,d) Cu@AlOx. The Cu (111) facet is used in all models.


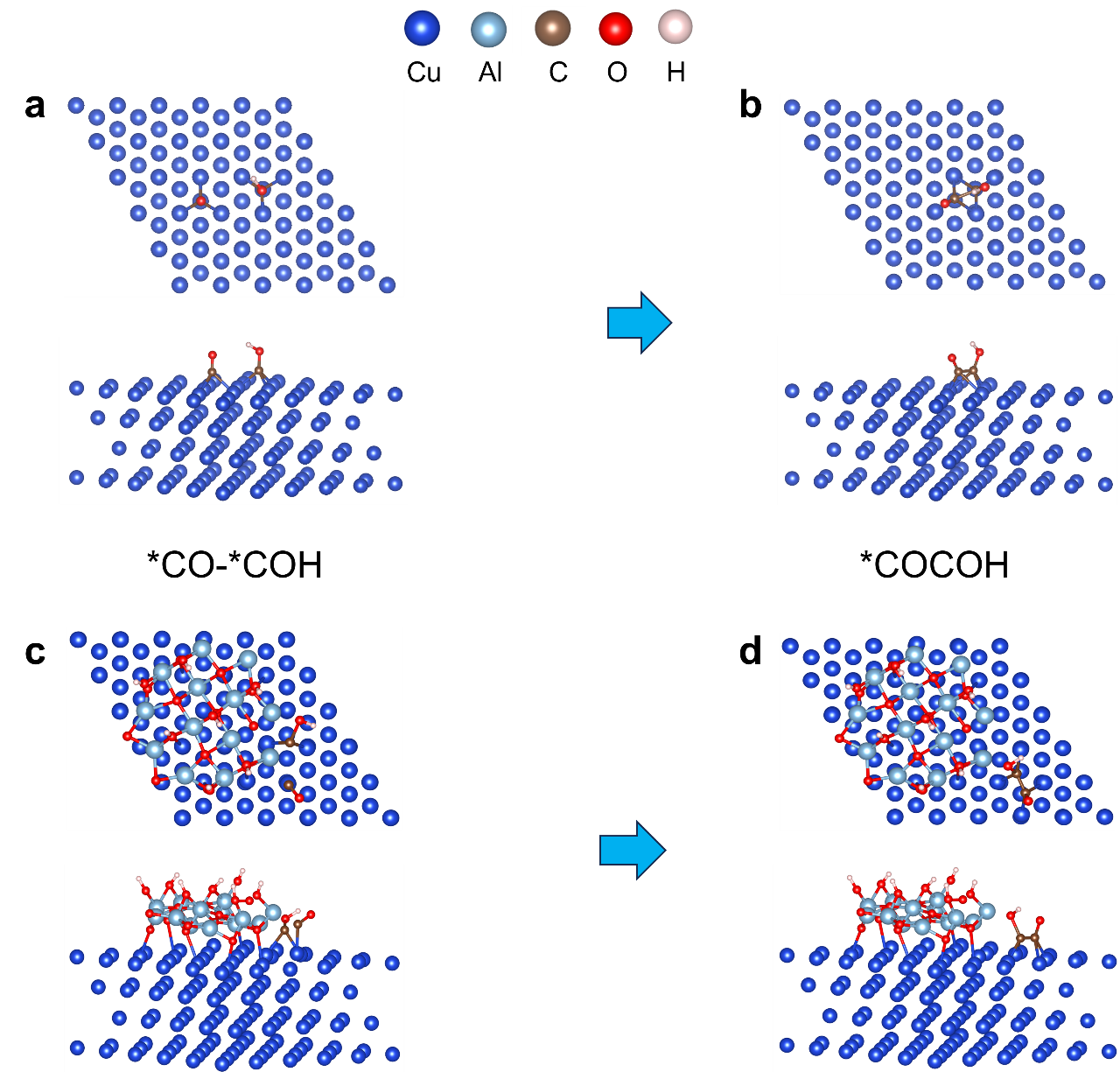


Figure S29. Top and side views of the DFT models showing conversion of *CO-*COH to *COCOH on (a,b) pristine Cu and (c,d) Cu @AlOx. The Cu (111) facet is used in all models.

**Table S1.** **Elemental composition of the Cu and Al in investigated samples determined by ICP-MS measurements. Cu/1000 indicates the amount of Cu diluted by 1000 times, and Al/10 indicates the amount of Al diluted by 10 times.**

| sample | Al/10  (ug L^-1^) | Cu/1000  (ug L^-1^) | experiment  n_Cu/Al_ | theory  n_Cu/Al_ |
| --- | --- | --- | --- | --- |
| Cu@AlOx_n=0_ | 33.5 | 27.1 | 34.5:1 | 30:1 |
| Cu@AlOx_n=0.5_ | 23.5 | 21.0 | 37.7:1 | 30:1 |

**Table S2. Physicochemical properties of Cu@AlOx NWs with the variation of the AA/AIP ratio.**

| Catalysts | Molar ratio of acetic acid to aluminum (AA/AIP) | S_BET_  (m^2^ g^–1^) | Average pore  size (nm) |
| --- | --- | --- | --- |
| Cu@AlOx_n=0_ | 0 | 48.22 | 2.6 |
| Cu@AlOx_n=0.5_ | 0.5 | 62.89 | 2.6 |

**Table S3.** **Comparison of all eCO_2_RR products between pristine Cu NWs and different Cu@AlOx NWs at different applied potentials.**

| **Catalyst** | **E *vs.* RHE**  **(V)** | **FE** | | | | | | | |
| --- | --- | --- | --- | --- | --- | --- | --- | --- | --- |
|  |  | H_2_ | CO | CH_4_ | C_2_H_4_ | HCOOH | C_2_H_5_OH | CH_3_COOH | n-C_3_H_7_OH |
| Cu NWs | -0.9 | 53.2±0.9 | 19.0 | 0.8±0.1 | 10.1±0.8 | 7.3±1.0 | 0.8±0.3 | 0 | 4.2±0.7 |
|  | -1.0 | 45.0±4.8 | 9.7±1.4 | 2.0±0.2 | 25.2±4.0 | 4.9±0.3 | 2.6±0.2 | 0.5±0.6 | 7.2±0.5 |
|  | -1.1 | 41.9±2.1 | 3.2±1.2 | 4.4±0.1 | 30.3±2.5 | 3.4±0.5 | 7.1±0.2 | 0.9±0.5 | 6.0±0.5 |
|  | -1.2 | 67.4±3.3 | 1.1±0.5 | 5.9±0.6 | 13.3±4.1 | 1.0±0.2 | 4.3±0.3 | 0.4±0.1 | 1.4±0.2 |
|  | -1.3 | 78.8±5.1 | 0.6±0.4 | 6.3±0.3 | 6.2±0.5 | 0.8±0.1 | 3.6±0.5 | 0 | 0 |
| Cu@AlO_n=0_ | -0.9 | 43.6±1.1 | 18.2±0.6 | 0.5±0.04 | 16.6±1.6 | 7.7±0.8 | 2.9±0.3 | 0 | 4.1±0.6 |
|  | -1.0 | 40.3±1.8 | 7.7±0.6 | 1.0±0.01 | 28.6±2.7 | 5.7±0.4 | 6.5±0.9 | 0.3±0.2 | 4.2±1.0 |
|  | -1.1 | 37.4±0.9 | 3.8±0.1 | 2.6±0.4 | 32.6±1.6 | 3.1±0.2 | 8.7±0.9 | 0.6±0.3 | 4.8±0.4 |
|  | -1.2 | 55.7±4.9 | 3.8±0.6 | 3.8±0.8 | 24.9±2.0 | 2.2±0.1 | 4.6±1.0 | 0.3±1.3 | 4.3±0.6 |
|  | -1.3 | 66.3±2.5 | 2.0±0.3 | 4.0±0.6 | 13.8±1.2 | 1.1±0.2 | 4.1±0.4 | 0.5±0.2 | 1.6±0.2 |
| Cu@AlO_n=0.5_ | -0.9 | 45.9±2.8 | 16.6±0.4 | 0.3±0.01 | 16.6±2.1 | 8.0±0.2 | 2.5±0.9 | 0 | 4.5±0.5 |
|  | -1.0 | 38.0±3.7 | 8.5±1.6 | 0.7±0.03 | 29.1±2.7 | 4.5±1.1 | 5.9±1.0 | 0.5±0.5 | 5.1±0.5 |
|  | -1.1 | 34.1±1.6 | 4.3±0.3 | 3.1±0.2 | 37.7±1.1 | 1.6±0.2 | 9.6±0.9 | 0.2±0.4 | 6.0±0.6 |
|  | -1.2 | 56.6±2.2 | 2.8±0.3 | 4.9±0.6 | 27.2±4.9 | 1.1±0.2 | 4.9±0.7 | 0.3±0.07 | 1.8±0.6 |
|  | -1.3 | 68.5±1.0 | 1.7±0.1 | 5.3±0.4 | 16.0±2.9 | 0.8±0.08 | 3.9±0.3 | 0.2±0.2 | 1.7±0.8 |

**Table S4. The ECSA of different catalysts before and after 48 h of eCO_2_RR measurements.**

| Catalysts | ECSA (cm^2^) | | |
| --- | --- | --- | --- |
|  | before eCO_2_RR | after eCO_2_RR | Rate of change (%)^a^ |
| Cu NWs | 48.9 | 95.6 | 95.3 |
| Cu@AlOx_n=0_ NWs | 46.6 | 74.9 | 57.8 |
| Cu@AlOx_n=0.5_ NWs | 40.7 | 62.5 | 44.5 |

^a^ The ECSA of pristine Cu NWs before eCO_2_RR is regarded as the baseline.

**Table S5. Comparison in eCO_2_RR performance between Al-modified Cu catalysts reported in previous literature and the Cu@AlOx_n=0.5_ NWs obtained in this work.**

| Catalyst | Electrolyzer | Electrolyte | E_RHE_  (V) | Current Density  (mA·cm^-2^) | FE_C1_  (%) | FE_C2+_  (%) | Stability | Ref. |
| --- | --- | --- | --- | --- | --- | --- | --- | --- |
| Cu_100_Al_7.88_-OD | flow cell | 1M KOH | -1.68 | 692.6^c^ | 8.4^b^ | 81.6^a^ | 8 h^a^ | Green Chem. 2024, 26, 2599. |
| CuAl_2_O_4_/CuO | flow cell | 1M KOH | 0.9 | 200^c^ | 7^b^ | 70^a^ | 150 h^a^ | Angew. Chem. 2023, 135, e202302096 |
| Cu NCs/ Al_2_O_3_-10c | flow cell | 5 M KOH | -1.1 | 300^c^ | ~13^b^ | 60.4^b^ | 24 h^a^ | Angew. Chem. 2021, 133, 25042 |
| Cu_2_O/Al_2_O_3_ | flow cell | 1.0 M KOH | 1.2 | 1200^c^ | ~5^b^ | 84.7^a^ | 82 h^a^ | Nano Energy 2025, 135, 110657. |
| CuAl -LDH | MEA | 0.1 M KHCO_3_ | -1.06 | ~50^c^ | 23^b^ | ~73^a^ | 11.1 h^a^ | Nano Research 2023, 16,4685. |
| Al-Cu/Cu_2_O | flow cell | 1 M KOH | -1.0 | 539.9^c^ | 8^b^ | 84.5^a^ | 20 h^a^ | J. Am. Chem. Soc. 2023, 145, 2194. |
| Cu_0.55_Al_0.45_O_x_ | flow cell | 1.0 M KOH | -0.55 | 50^c^ | ~10 ^b^ | ∼28^b^ | N.A. | J. Am. Chem. Soc. 2023, 145, 6953. |
| Cu/P-Al_2_O_3_ SAC | flow cell | 1.0 M KOH | -1.2 | 152.9^c^ | 82^b^ | 5^b^ | 20 min^b^ | Nano Lett. 2021, 21, 7325 |
| Cu-CuAlO_2_-Al_2_O_3_ | H-cell | 0.1 M KHCO_3_ | -1.2 | 61.1^c^ | 6.5^b^ | 85.6^a^ | 300 h^a^ | J. Am. Chem. Soc. 2022, 144, 22759. |
| Cuo/Al_2_CuO_4_ | H-cell | 0.1 M KHCO_3_ | -0.99 | 3^c^ | ~3^b^ | 82.4^a^ | 100 h^a^ | Energy Environ. Sci. 2022, 15, 2397. |
| Cu@AlOx_n=17_ NCs | H-cell | 0.1 M KHCO_3_ | -1.1 | 2.7^c^ | 46.8^a^ | 28.2^a^ | 24 h^a^ | Nat. Mater.  2024, 23, 680. |
| Cu@AlOx_90_ | H-cell | 0.1M KHCO_3_ | -1.2 | ~3.2^c^ | ~53^b^ | ~14^b^ | 15 h^a^ | J. Am. Chem. Soc. 2025, 147, 29, 25517. |
| Cu@AlOx_n=0.5_ NWs | **H cell** | **0.1 M KHCO_3_** | **-1.1** | **60** | **9** | **53.5** | **48 h** | **This work** |
|  | **flow cell** | **1.0 M KOH** | **-0.83**  **(600 mA)** | **600** | **14.0** | **69.6** | **64h** |  |

^a^ The data is showed in the literature. ^b^ The data is estimated from the figures in the literature.

^c^ The current density is normalized to the geometric area.

**Table S6. The binding energy of Cu 2p in pristine Cu NWs, Cu@AlOx_n=0.5_ NWs, and Cu@AlOx_n=0_ NWs before and after 24 h of eCO_2_RR measurements.**

| Catalysts | before eCO_2_RR | after eCO_2_RR |
| --- | --- | --- |
| Cu NWs | 932.66 eV | 932.59 eV |
| Cu@AlOx_n=0_ NWs | 932.76 eV | 932.71 eV |
| Cu@AlOx_n=0.5_ NWs | 932.78 eV | 932.71 eV |

**Table S7. The valence state variation of Cu in pristine Cu NWs, Cu@AlOx_n=0.5_ NWs, and Cu@AlOx_n=0_ NWs before and after 24 h of eCO_2_RR measurements.**

| Sample | Cu^2+^ (%) | Cu^+^ (%) | Cu^0^ (%) |
| --- | --- | --- | --- |
| Cu NW  Before eCO_2_RR | 0 | 83.0 | 17.0 |
| Cu NW  After eCO_2_RR | 0 | 31.2 | 69.8 |
| Cu@AlOx_n=0_  Before eCO_2_RR | 70.8 | 29.2 | 0 |
| Cu@AlOx_n=0_  After eCO_2_RR | 3.7 | 82.9 | 13.4 |
| Cu@AlOx_n=0.5_  Before eCO_2_RR | 74.0 | 26.0 | 0 |
| Cu@AlOx_n=0.5_  After eCO_2_RR | 2.6 | 80.9 | 16.5 |

**Table S8. XPS comparison of Al 2p and Cu 3p region for Cu@AlOx_n=0_ NWs and Cu@AlOx_n=0.5_ NWs before and after 24 h of eCO_2_RR measurements at -1.1 V *vs.* RHE.**

| Sample | Region | Binding Energy  (eV) | FWHM  (eV) | Area | Area ratio (%) |
| --- | --- | --- | --- | --- | --- |
| Cu@AlOx_n=0_  Before CO_2_RR | Cu 3p  Al 2p | 77.10/79.14  74.86 | 2.5  2.7 | 86594.2  95557.8 | 47.5  52.5 |
| Cu@AlOx_n=0_  After CO_2_RR | Cu 3p  Al 2p | 77.33/79.32  75.04 | 2.7  2.9 | 14172.8  12253.3 | 53.6  46.4 |
| Cu@AlOx_n=0.5_  Before CO_2_RR | Cu 3p  Al 2p | 77.05/79.01  75.08 | 2.5  2.4 | 95570.5  108279.6 | 46.9  53.1 |
| Cu@AlOx_n=0.5_  After CO_2_RR | Cu 3p  Al 2p | 77.32/79.32  75.13 | 2.3  2.5 | 12688.0  11033.8 | 53.5  46.5 |

| Catalysts | before eCO_2_RR | | | after eCO_2_RR | | | | | | | | |
| --- | --- | --- | --- | --- | --- | --- | --- | --- | --- | --- | --- | --- |
|  | 0 h | | | 24 h | | | 48 h | | | 72 h | | |
|  | c_Cu_  (mg  L^-1^) | c_Al_  (µg  L^-1^) | n_Cu_:n_Al_ | c_Cu_  (mg L^-1^) | c_Al_  (µg  L^-1^) | n_Cu_:n_Al_ | c_Cu_  (mg L^-1^) | c_Al_  (µg L^-1^) | n_Cu_:n_Al_ | c_Cu_  (mg L^-1^) | c_Al_  (µg L^-1^) | n_Cu_:n_Al_ |
| Cu NWs | 27.0 |  |  | 26.4 |  |  | 26.2 |  |  | 24.0 |  |  |
| Cu@AlOx_n=0_ NWs | 27.4 | 335.5 | 34.5:1 | 25.5 | 74.8 | 143.6:1 | 13.7 | 24.4 | 236.1:1 | 16.2 | 24.7 | 276.1:1 |
| Cu@AlOx_n=0.5_ NWs | 21.0 | 234.8 | 37.7:1 | 28.9 | 105.9 | 115.0:1 | 33.8 | 11.9 | 128.6:1 | 30.9 | 71.4 | 182.6:1 |

**Table S9.** **Elemental composition of the Cu and Al in investigated samples before and after eCO_2_RR measurements determined by ICP-MS.**

**References:**

[1] C.-J. Chang, S.-C. Lin, H.-C. Chen, J. Wang, K.J. Zheng, Y. Zhu, H.M. Chen, *J. Am. Chem. Soc.* **2020,** *142*, 12119-12132.

[2] C. Choi, S. Kwon, T. Cheng, M. Xu, P. Tieu, C. Lee, J. Cai, H.M. Lee, X. Pan, X. Duan, W.A. Goddard, Y. Huang, *Nat. Catal.* **2020,** *3*, 804-812.

[3] P.P. Albertini, M.A. Newton, M. Wang, O. Segura Lecina, P.B. Green, D.C. Stoian, E. Oveisi, A. Loiudice, R. Buonsanti, *Nat. Mater.* **2024,** *23*, 680-687.

[4] S.-M. Kim, Y.-J. Lee, J.W. Bae, H.S. Potdar, K.-W. Jun, *Appl. Catal. A Gen.* **2008,** *348*, 113-120.

[5] H. Li, P. Yu, R. Lei, F. Yang, P. Wen, X. Ma, G. Zeng, J. Guo, F.M. Toma, Y. Qiu, S.M. Geyer, X. Wang, T. Cheng, W.S. Drisdell, *Angew. Chem. Int. Ed.* **2021,** *60*, 24838-24843.

[6] Y. Sha, J. Zhang, X. Cheng, M. Xu, Z. Su, Y. Wang, J. Hu, B. Han, L. Zheng, *Angew. Chem. Int. Ed.* **2022,** *61*, e202200039-e202200044.

[7] S. González, M. Pérez, M. Barrera, A.R. González Elipe, R.M. Souto, *J. Phys. Chem. B* **1998,** *102*, 5483-5489.

[8] L. Wang, K. Gupta, J.B.M. Goodall, J.A. Darr, K.B. Holt, *Faraday Discuss.* **2017,** *197*, 517-532.

[9] S. Gao, Y. Lin, X. Jiao, Y. Sun, Q. Luo, W. Zhang, D. Li, J. Yang, Y. Xie, *Nature* **2016,** *529*, 68-71.

[10] D. Voiry, M. Chhowalla, Y. Gogotsi, N.A. Kotov, Y. Li, R.M. Penner, R.E. Schaak, P.S. Weiss, *ACS Nano* **2018,** *12*, 9635-9638.

[11] S. Trasatti, O.A. Petrii, *J. Electroanal. Chem.* **1992,** *327*, 353-376.

[12] G. Kresse, J. Hafner, *Phys. Rev. B* **1993,** *48*, 13115-13118.

[13] G. Kresse, J. Furthmüller, *Phys. Rev. B* **1996,** *54*, 11169-11186.

[14] J.P. Perdew, K. Burke, M. Ernzerhof, *Phys. Rev. Lett.* **1996,** *77*, 3865-3868.

[15] W. Kohn, L.J. Sham, *Phys. Rev.* **1965,** *140*, A1133-A1138.

[16] S. Grimme, J. Antony, S. Ehrlich, H. Krieg, *J. Chem. Phys.* **2010,** *132*, 154104.

[17] V. Wang, N. Xu, J.-C. Liu, G. Tang, W.-T. Geng, *Comput. Phys. Commun.* **2021,** *267*, 108033.

[18] K. Momma, F. Izumi, *J. Appl. Crystallogr.* **2008,** *41*, 653-658.
